# Exosome microRNA-22 inhibiting proliferation, migration and invasion through regulating Twist1/CADM1 axis in osteosarcoma

**DOI:** 10.1038/s41598-023-50612-4

**Published:** 2024-01-08

**Authors:** Qing Ruan, Cuijie Wang, Yuntao Wu, Qingsan Zhu

**Affiliations:** 1 Department of Orthopedics of the China-Japan Union Hospital of Jilin University, Sendai Street 126, Changchun, 130033 Jilin China; 2 Department of Anesthesiology of the China-Japan Union Hospital of Jilin University, Sendai Street 126, Changchun, 130033 Jilin China

**Keywords:** Cancer, Cell biology

## Abstract

This study aims to the function of miR-22 original mesenchymal stem cells (MSC) on osteosarcoma (OS) proliferation, migration and invasion. Bio-informatics analysis including GEO2R analysis, Gene Ontology analysis, integration analysis were used to confirmed the target genes (miR-22, Twist1, CADM1) in OS. RT-qPCR and western blotting confirmed the different expression of miR-22, Twist1, CADM1 in OS tissues, MG63 and Saos cell lines. MTS assay, CCK8 assay, colony forming assay, EdU assay were performed to detect the proliferation effect of miR-22 on MG63. Transwell migration assay, transwell invasion assay, wound healing assay were used to verify the migration and invasion effect of miR-22 on MG63. Luciferase reporter assay confirm the binding sites between miR-22 and Twist1. RT-qPCR confirmed miR-22 and CADM1 downregulated and Twist1 upregulated in OS tissues, MG63 and Saos. Exosome original MSC labeled with PKH-26 could be uptake by MG63, which upregulated the expression of miR-22 in MG63. High expression of miR-22 in MG63 inhibited proliferation, migration and invasion, which could be rescued by Twist1. Dual luciferase reporter analysis confirmed Twist1 was a target of miR-22. Exosome modified with miR-22 mimic inhibit proliferation, migration and invasion more efficient than exosome original MSC. miR-22 cargo in exo-MSC could uptake by MG63 and supply MG63 with miR-22, which inhibit MG63 proliferation, migration and invasion through targeting Twist1.

## Introduction

Osteosarcoma (OS) is a primary malignant tumor that has a tendency to occur in adolescents. The most common symptom is local pain at the epiphyseal end of the long shaft. The incidence rate of OS in adolescents is 8–11/million/year^1–3^. At present, the main clinical treatment method for OS is surgery combined with radiotherapy and chemotherapy, but the outcomes and prognosis is disappointing, especially for patients with lung metastasis^4^. The pathogenesis of the OS is still unclear. Therefore, finding the key genes and signaling pathways involved in the OS are imminent^5^.

In 2010, therapeutic mesenchymal stem cell exosomes (exo-MSC) were first described, which has effects on repairing liver fibrosis^6^, retinal laser injury^7^, limb ischemia injury^8^, diabetes induced cognitive injury^9^ and inflammation induced premature brain injury^10^. At the same time, it can also inhibit the metastasis of breast cancer cells^11^, promote tumor apoptosis, inhibit tumor proliferation. These anti-tumor effect of exo-MSC mainly attribute to the cargo in exo-MSC, which including lipids, proteins and nucleic acids.

MicroRNAs (miRNA) are main cargo in exo-MSC, which participated in many disease including OS. miR-22 is an evolutionarily conservative gene located on chromosome 17p13. The promoter transcription start site lacks a TATA box^12^. miR-22 is abnormally up-regulated or down-regulated in many cancers, such as prostate cancer, esophageal squamous cell carcinoma, breast cancer and gastric cancer^13–16^. It not only serves as as a tumor suppressor miRNA, but also as a carcinogenic miRNA that hinders or exacerbates cancer formation and malignant transformation. Due to the complexity of the regulatory mechanisms and the lack of satisfactory elucidation, the potential mechanisms and clinical applications of miR-22 in regulating OS progression have not been elucidated.

miR-22 may significantly affect the biological behavior of cancer, such as the proliferation, invasion, and metastasis, and it genetically alters the expression of many related genes. Studies confirmed that miR-22 targets and inhibits downstream transcription factors, could determine the fate of cancer. Twist1 is one of the transcription factors targeted by miR-22^17^. Twist1 is first discovered in fruit flies and has gradually been found to play an important role in embryonic development and pathological diseases. The Twist1 gene is located on chromosome 7p21 and contains two exons and one intron. It reports to closely relate to tumor metastasis^18^. At present, the expression of Twist1 is abnormally high expression in many tumors, such as breast cancer, prostate cancer, hepatocellular carcinoma. Twist1 plays multiple roles in cancer, affecting the occurrence, progression, and metastasis of tumors. Targeting treatment of Twist1 or Twist1 related molecules has shown to be a promising cancer therapy method.

In this study, we performed bio-informatics analysis to identify the potential roles of miRNA-22/Twist1/CADM1 in OS pathogenesis. Verification experiments confirmed the function of miRNA-22 on proliferation, migration and invasion in OS. Exosomes were acquired from MSC medium supernatant, and modified with miRNA-22 mimic and inhibitor. The function of exo-MSC with high expression of miRNA-22 on MG63 proliferation, migration and invasion were also verified.

## Materials and methods

### Tissue collection

This study was conducted in accordance with the Declaration of Helsinki and was approved by the Ethics Committee of the China-Japan Union Hospital of Jilin University. The tumor tissues and normal control were obtained from OS patients (n = 3). All patients agreed to the use of their samples in scientific research and provided written informed consent.

### Bioinformatic analysis

Sequencing results of OS were downloaded from GEO datasets (https://www.ncbi.nlm.nih.gov/gds). Funrich analysis was executed by Venn (http://bioinformatics.psb.ugent.be/webtools/Venn/). Sangerbox was used for visualization processing (http://sangerbox.com/). Targetscan (targetscan.org/) was used to predict the binding sites between miRNAs and mRNA. EVmiRNA (http://bioinfo.life.hust.edu.cn/EVmiRNA/#!/) was used to predict the amount of miRNA cargo in exo-MSC. Gene Ontology analysis was carried by DAVID (https://david.ncifcrf.gov/summary.jsp).

### Real-time quantitative PCR (RT-qPCR)

The total RNA was extracted using TRIzol® reagent (Invitrogen; Thermo Fisher Scientific, Inc.). Total RNA was reverse-transcribed to cDNA using PrimeScript RT reagent kit (Takara Bio, Inc.). RT-qPCR was performed to confirm the relative expression of miRNA and mRNA. The relative expression of gene was calculated by the 2^-ΔΔ Ct^ method after normalized to GAPDH or U6. The primers were as follows: Twist1 (forward) 5′-GTCCGCAGTCTTACGAGGAG-3′, Twist1 (reverse) 5′-GCTTGAGGGTCTGAATCTTGCT-3′; CADM1 (forward) 5′-ATGGCGAGTGTAGTGCTGC-3′, CADM1 (reverse) 5′-GATCACTGTCACGTCTTTCGT-3′; MMP2 (forward) 5′-AGGATGGCAAGTACGGCTTC-3′, MMP2 (reverse) 5′-AGCTGTTGTAGGATGTGCCC-3′; PCNA (forward) 5′-TGGCGTGAACCTCACCAG-3′, PCNA (reverse) 5′-GGAGACAGTGAAGTGGCTTTTG-3′; GAPDH (forward) 5′-CGGACCAATACGACCAAATCCG′, GAPDH (reverse) 5′-AGCCACATCGCTCAGACACC-3′; miR-22 (forward) 5′-GCTACTCGAGTCCCCCGCTCATCTAGA-3′, miR-22 (reverse) 5′-GCCAAGCTTCGAGGGGGAGCAAATCAC-3′; U6 (forward), 5′-CTCGCTTCGGCAGCACA-3′, U6 (reverse), 5′-AACGCTTCACGAATTTGCGT-3′.

### Cell culture

MSC, MG63, Saos were purchased from the Institute of Biochemistry and Cell Biology and cultured in DMEM (Gibco; Thermo Fisher Scientific, Inc.) in 10% fetal bovine serum at 37 °C in a humidified 5% CO2 incubator. The medium was replaced every 1–2 days. Exosomes (50 ug/ml) was added into the culture medium for co-culture experiments.

### Cell transfection

miR-22 mimics and pcDNA3.1-Twist1 vector were used to overexpression of miR-22 and Twist1, respectively. miR-22 inhibitor and Twist1 siRNA (si-Twist1) were used to downexpression of miR-22 and Twist1, respectively. All the RNAi were designed and synthesized by GenePharma (Shanghai, China). Lipofectamine® 2000 (Invitrogen; Thermo Fisher Scientific, Inc.) with 250 pmol mimic or inhibitor were transfected into MSC or MG63 about 5 h at 37 °C. Total miRNAs, mRNAs and proteins were harvested after 48 h.

### Western blotting analysis

The protein was extracted using RIPA buffer (Beijing Solarbio Science & Technology Co., Ltd.) and quantified using a BCA assay (Thermo Fisher Scientific, Inc.). Total cell protein content (30 µg/lane) was isolated by 10% SDS-PAGE (cat. no. 1200; Beijing Solarbio Science & Technology Co., Ltd.), and transferred to nitrocellulose membrane (0.45 μm; EMD Millipore). Blots were blocked with 5% nonfat milk with 0.1% Tween 20 (Sigma-Aldrich; Merck KGaA) for 2 h at 37 °C. followed by secondary antibodies GAPDH at room temperature for 50 min.

### Dual luciferase reporter

The wild or mutant type Twist1 3′ -URT were cloned into pGL6- miR vector (Beyotime, China). 2 × 10^4^ MG63 cells were seeded into 6-well plates for 24 h at 37 °C before transfection. Luciferase kit (Beyotime) was used after pGL6-Twist1-wt, pGL6-Twist1-mut and miR-22 mimics as well as mimic controls co-transfected into MG63 cells with Lipofectamine 2000 (Invitrogen, Life Technologies). At 24 h post-transfection, the luciferase were measured at 560 nm.

### Proliferation

The proliferation of MG63 transfected with miR-22 mimics or inhibitor, Twist1 vector or Twist1 siRNA was determined by the Cell Counting Kit-8 (CCK-8) assay, colony formation assay and EdU assay according to the manufacturer's instructions. CCK -8 assay: A density of 2 × 10^4^ cells were seeded in 96-well plate for 12–24 h. 10 µl CCK solution was putted into medium and incubated for 2 h 37 °C. The absorbance at 450 nm was detected by an Epoch microplate (Bio-Tek Instruments). Colony formation assay: The cells were plated in 6-well plates at 200 per well after transfection. After 2 weeks, the cells were washed twice with PBS, fixed with methanol and stained with 0.5% crystal violet. The number of colonies was counted under a microscope. EdU assay: Cells were cultured in the EdU solution (50 um) for 2 h. Cells were treated by 4% paraformaldehyde for 15 min, 0.3% Triton X-100 for 20 min, Click Reaction liquid for 30 min, DAPI for 10 min, and measured by a confocal microscope.

### Migration and invasion assays

The migration and invasion of MG63 transfected with miR-22 mimics or inhibitor, Twist1 vector or Twist1 siRNA were determined by transwell assay, wound healing assay. For the cell migration and invasion assay, 10^4^ MG63 cells in serum-free medium were placed into the upper chamber of a 24-well Transwell Chamber (8 μm pore size, Corning Costar Corporation, Cambridge, MA, USA) uncoated or coated with Matrigel (BD Biosciences, San Jose, CA, USA). The chambers were incubated for 24 h in culture medium containing 10% FBS added to the lower chamber. The non-invaded cells were removed with cotton swabs. Cells invading to the lower surface were fixed, stained and counted using an inverted microscope. For the wound healing assay, create one straight-line scratches using a 200 uL pipette at the centre of the 6-well plate, and then washed with PBS to remove floating cells and cell debris. Cells were stimulated in DMEM plus serum for 24 h. The scratch boundaries were imaged under an inverted microscope and analysed using the Image J software.

### Isolation of exosomes from culture medium

Culture medium was centrifuged at 300 g for 10 min, 2000 g for 20 min, 10,000 g for 30 min, and 100,000 g for 90 min, re-suspended, and centrifuged at 100,000 g for 90 min.

### Transmission electron microscopy

Exosome were fixed in 2% glutaraldehyde and postfixed in 1% osmium tetroxide. Ultrathin sections were cut and stained with 10% uranyl acetate and 1% lead citrate. The ultrastructure of exosome was observed by using a transmission electron microscope (HITACHI H-7000, Tokyo, Japan).

### Fluorescent labeling of exosomes

Exosomes derived from MSCs were labeled with lipophilic dye PKH26 liquor (MINI26-1KT, Sigma, USA). Briefly, exosomes resuspended by 25 ul PBS was mixed with 1 ml dilution C. At the same time, 4 ul PKH26 was mixed with 1 ml dilution C. then these two mixtures were blended together and incubator at 37 °C with 5% CO2 for 3 min. 2 ml FBS was added into the mixed liquid to termination the reaction. Finally, the labeled exosomes were centrifuged at 100,000 g for 70 min and rinsed by 5 ml PBS. 1 ml PBS with exosomes was added into the MG63 medium supernatant and co-culture for 2 h. The MG63 was washed by PBS twice and stained with 4,6-diamidino-2-phenylindole (DAPI). At last, internalization of exosomes was measured by a confocal microscope.

### Statistical analysis

All statistical analyses were performed by SPSS 20.0 (IBM Corp.). The data are presented as the mean ± standard deviation. Comparisons among groups were analyzed using one-way ANOVA followed by Bonferroni's post hoc test. *p* < 0.05 was considered to indicate statistically significant.

## Results

### miR-22, Twist1 and CADM1 are involved in OS pathogenesis

**Figure 1:**
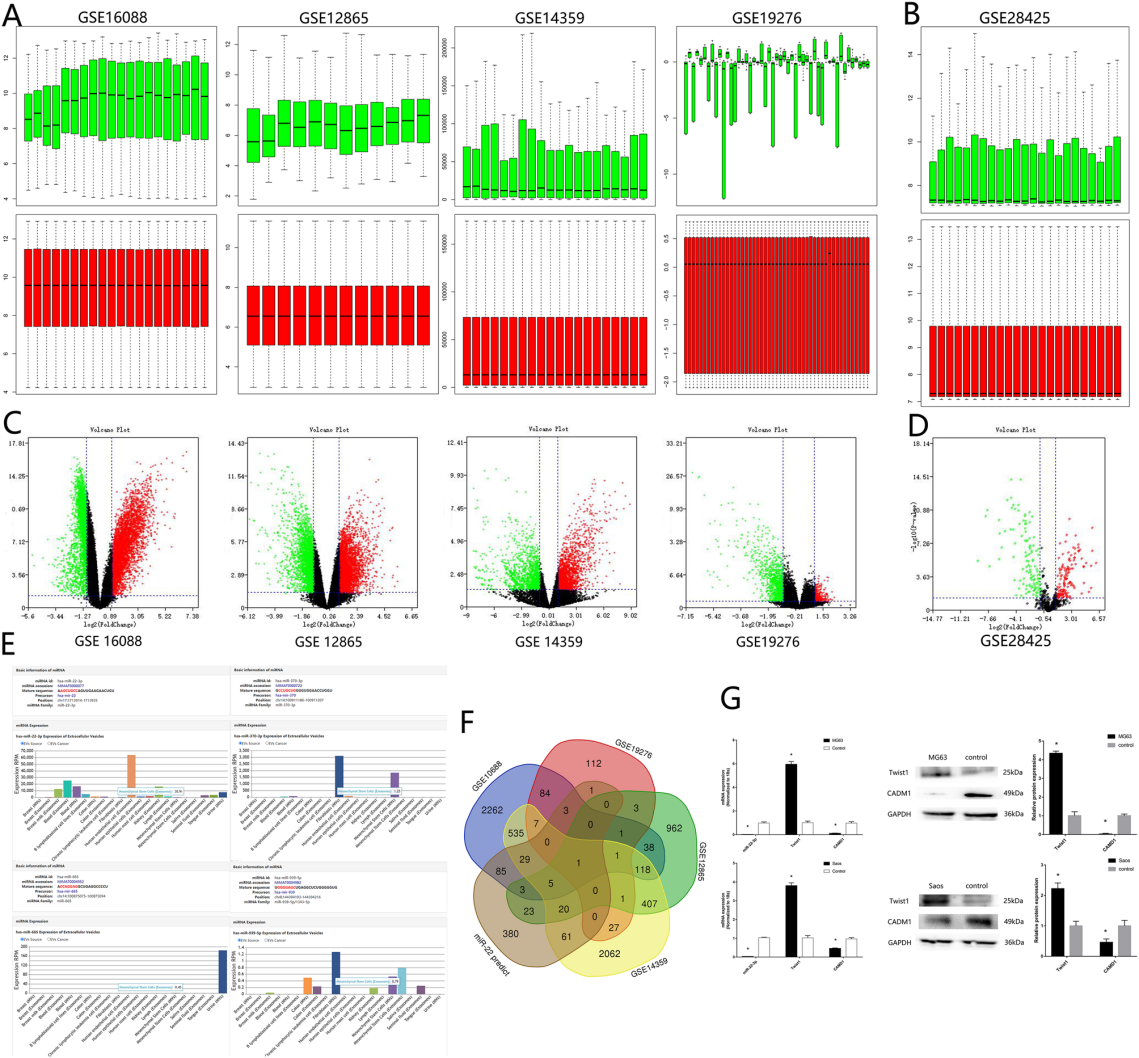
miR-22, Twist1 and CADM1 are involved in OS pathogenesis. (**A**, **B**) Homogenization by R language. (**C**, **D**) Volcano plot. (**E**) EVmiRNA bioinformation analysis. (**F**) Venn analysis. (**G**) RT-qPCR and western blotting verify the expression of miR-22, Twist1 and CADM1 (n = 3, *P ≤ 0.05 vs. control).

4 OS mRNA sequencing data (GSE16088, GSE12865, GSE14359, GSE19276) and 1 OS miRNA sequencing data GSE28425 were downloaded from GEO databases. After homogenization analysis (Fig. 1A,B), different expression genes were showed as volcano plot (Fig. 1C,D). There are 116 different down expression miRNA in GSE28425. EVmiRNA bioinformation analysis showed that miR-22 was the highest cargo in exo-MSC comparing with other 115 miRNA in GSE28425 (Fig. 1E). Targetscan showed that there were 611 target genes which had binding sites with miR-22. Venn analysis showed that CADM1 was the only 1 different expression gene (Fig. 1F). Gene Ontology analysis showed that epithelial mesenchymal transition (EMT) was the relate signal pathway during OS. ZEB1, Snail, and Twist1 were transcription factors related to the EMT process, which could inhibit the transcription of E-cadherin. Twist1 was the only 1 transcription factor which had binding sites with both miR-22 and CADM1. Then we predicted miR-22, Twist1 and CADM1 might participate in the OS. RT-qPCR showed a decreased expression of miR-22 in OS patients tissues, MG63 and Saos cells lines comparing with corresponding non-neoplastic normal tissues and MSC cell line (Fig. 1G). RT-qPCR and western blotting analysis showed high expression of Twist1 and low expression of CAMD1 in OS patients tissues, MG63 and Saos cells lines (Fig. 1G). These results showed that miR-22 is involved in OS pathogenesis.

### Downregulation of miR-22 regulate MG63 proliferative, migration and invasion

**Figure 2:**
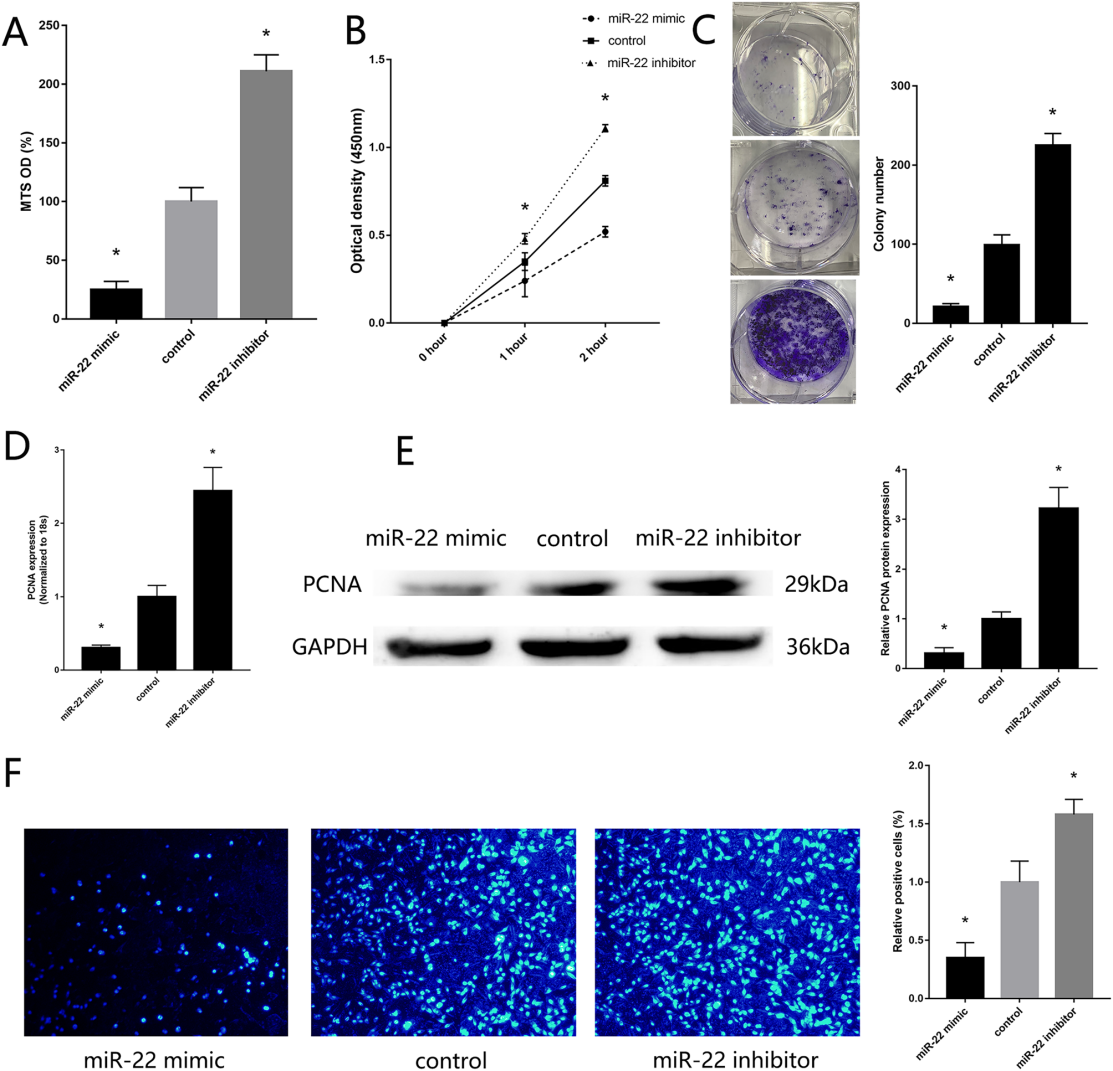
Downregulation of miR-22 regulates MG63 proliferative. (**A**) MTS assay detected the proliferation of MG63 transfected with miR-22 mimic and inhibitor. (**B**) CCK-8 assay. (**C**) Colony formation assay. (**D**) RT-qPCR detected the expression of PCNA. (**E**) Western blotting detected the expression of PCNA. (F) EdU assays. (n = 3,*P ≤ 0.05 vs. control).

**Figure 3:**
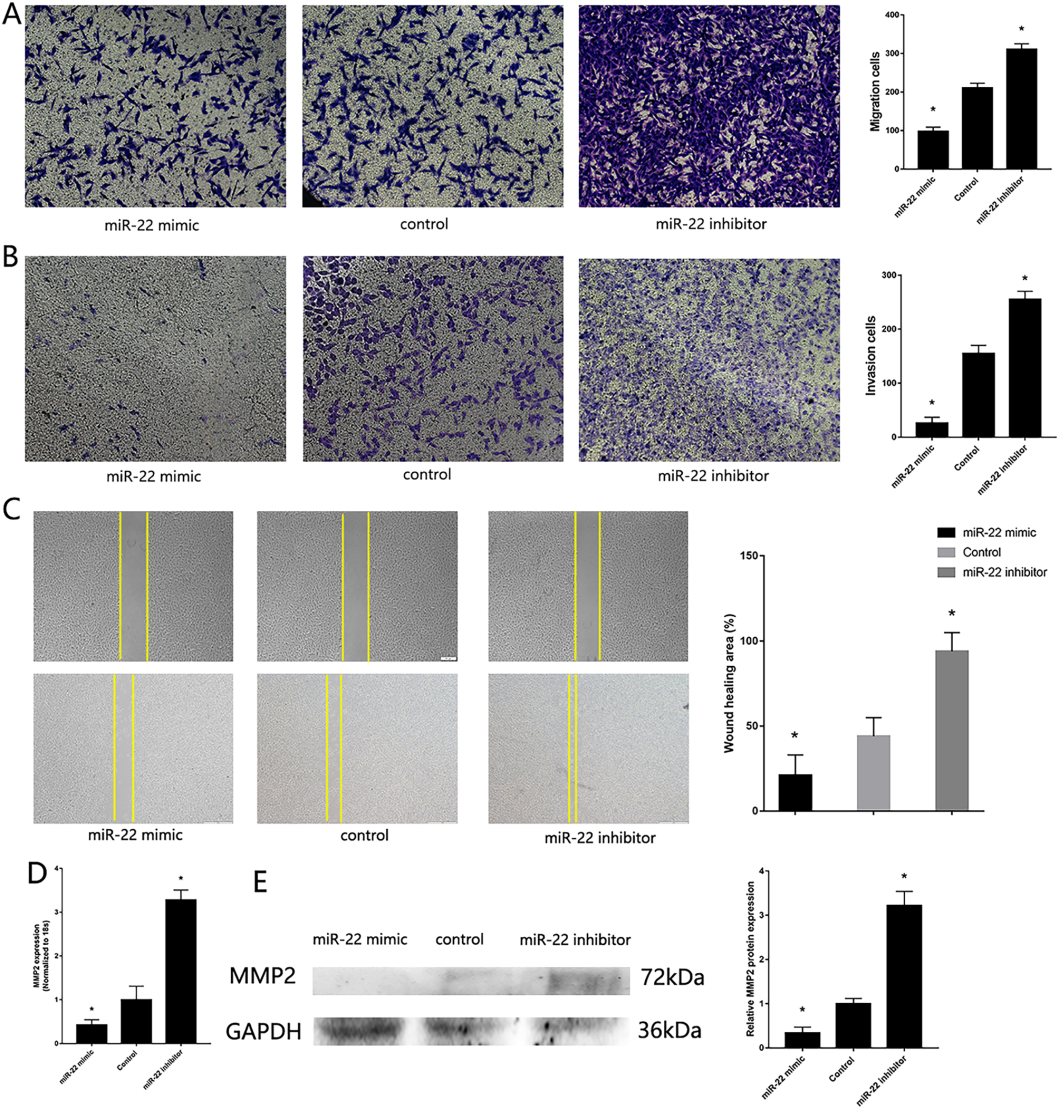
Downregulation of miR-22 regulates MG63 *migration and invasion*. (**A**) Transwell migration assay detected the migration of MG63 transfected with miR-22 mimic and inhibitor. (**B**) Transwell invasion assay detected the invasion of MG63 transfected with miR-22 mimic and inhibitor. (**C**) Wound healing assay. (**D**) RT-qPCR detected the expression of MMP2. (**E**) Western blotting detected the expression of MMP2 (n = 3,*P ≤ 0.05 vs. control).

To verify the previous finding of miR-22 participated in the regulation of OS pathogenesis, we transfected miR-22 mimic and inhibitor into MG63. PCR and western blotting confirmed the transfected efficiency. High expression of miR-22 was found in MG63 transfected with miR-22 mimic, down expression of Twist1 and high expression of CADM1 were found in MG63 transfected with miR-22 mimic (Supplement Fig. 1A). MTS assay (Fig. 2A), CCK-8 assay (Fig. 2B) and colony formation assay (Fig. 2C) showed upregulated proliferative of MG63 with low expression of miR-22, downregulated proliferative of MG63 with high expression of miR-22. RT-qPCR (Fig. 2D) and western blotting (Fig. 2E) showed high expression of PCNA in MG63 with low expression of miR-22. miR-22 mimic reduced cell proliferation, as confirming by EdU proliferation assays (Fig. 2F). Transwell assay, wound healing assay showed downregulated migration and invasion of MG63 with high expression of miR-22 (Fig. 3 A-C). RT-qPCR (Fig. 3D) and western blotting (Fig. 3E) showed up-regulated expression of MMP2 in MG63 with low expression of miR-22. These results showed that miR-22 inhibit MG63 proliferative, migration and invasion.

### miR-22/Twist1 regulates MG63 proliferative, migration and invasion

**Figure 4:**
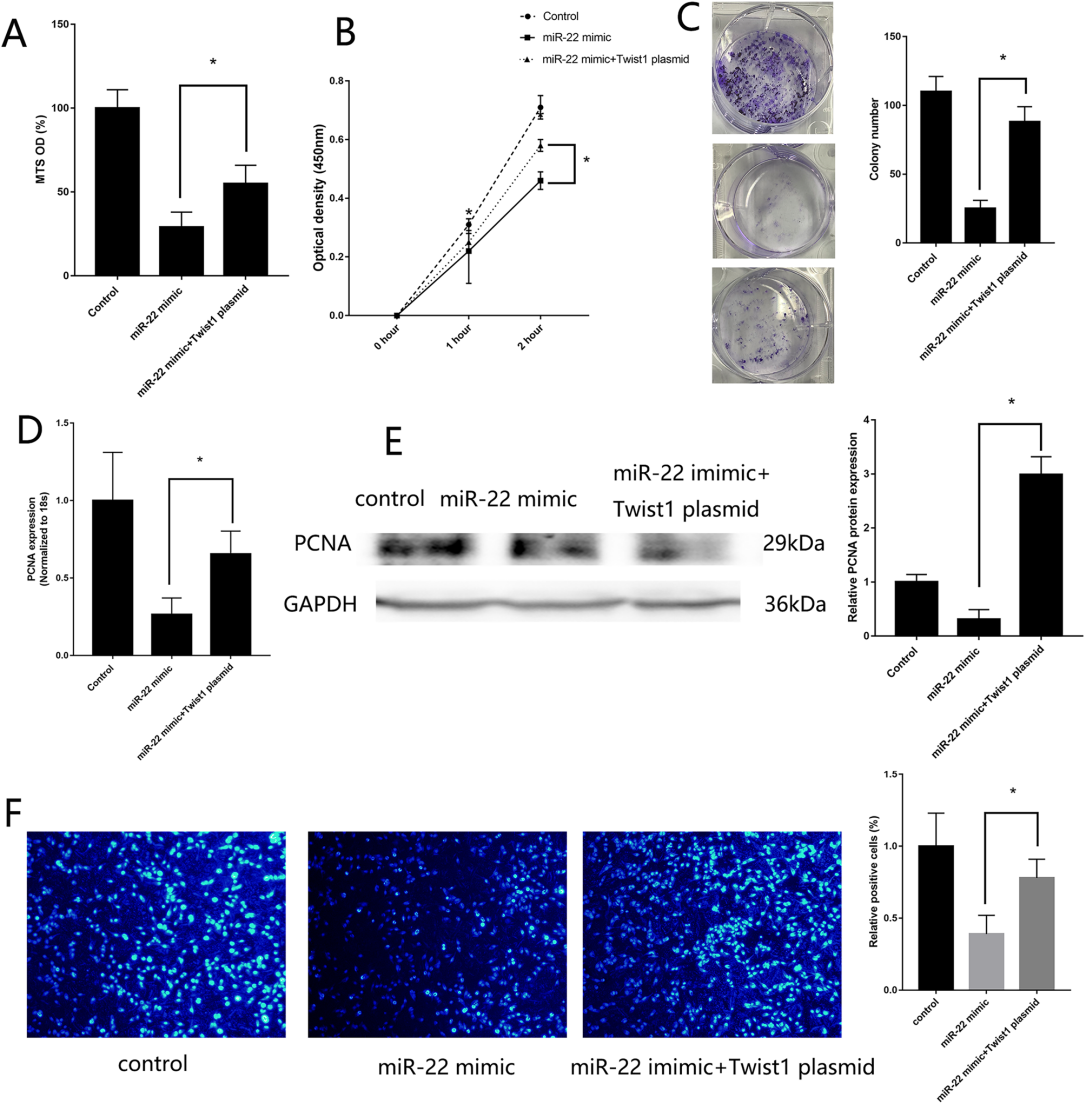
*miR-22/Twist1 regulates MG63 proliferation*. (**A**) MTS assay detected the proliferation of MG63 transfected with both miR-22 mimic and Twist1 vector. (**B**) CCK-8 assay. (**C**) colony formation assay. (**D**) RT-qPCR detected the expression of PCNA. (**E**) Western blotting detected the expression of PCNA. (**F**) EdU assays. (n = 3,*P ≤ 0.05 vs. control).

**Figure 5:**
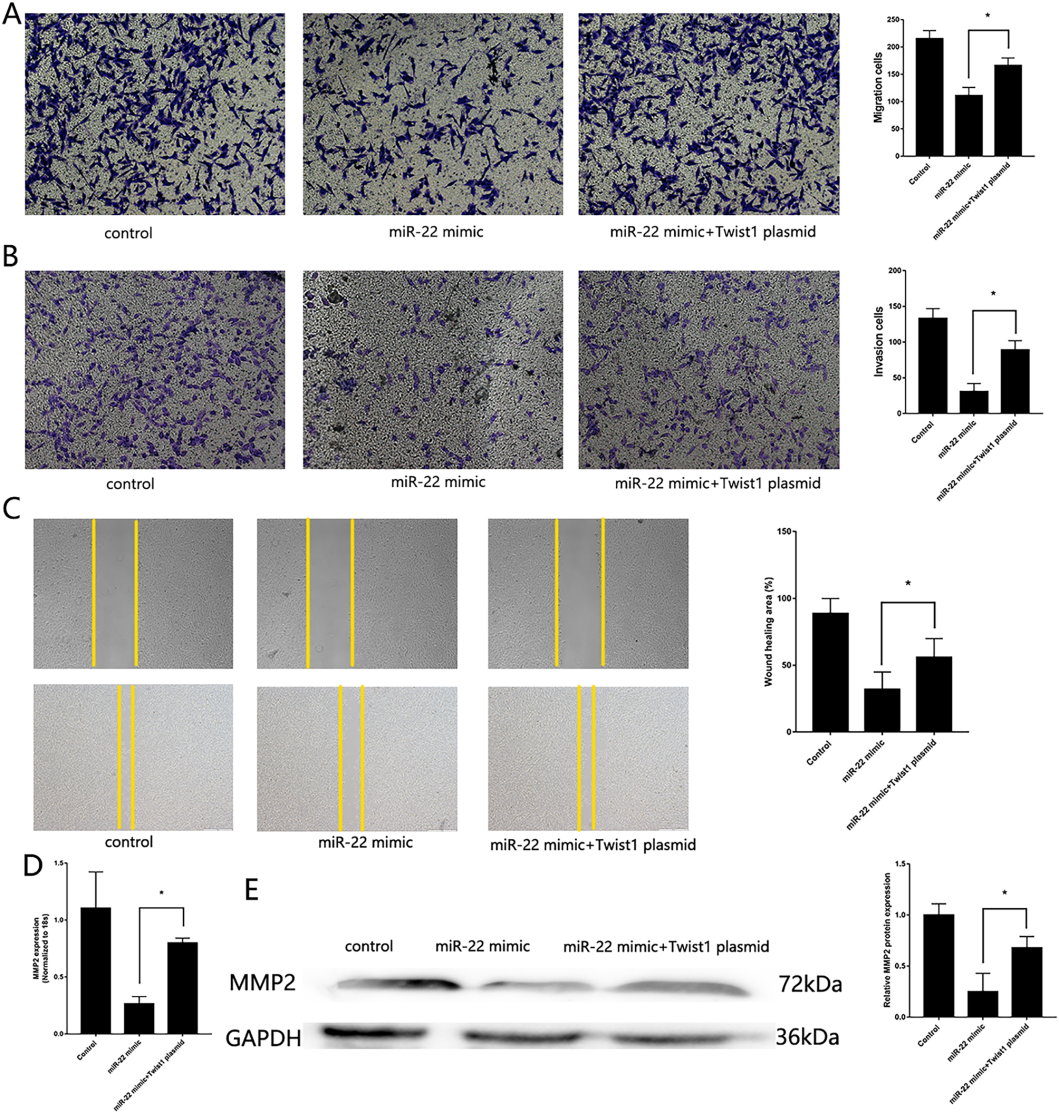
*miR-22/Twist1 regulates MG63 migration and invasion*. (**A**) Transwell migration assay detected the migration of MG63 transfected with both miR-22 mimic and Twist1 vector. (**B**) Transwell invasion assay detected the invasion of MG63 transfected with both miR-22 mimic and Twist1 vector. (**C**) Wound healing assay. (**D**) RT-qPCR detected the expression of MMP2. (**E**) Western blotting detected the expression of MMP2 (n = 3,*P ≤ 0.05 vs. control).

The transfection efficiency of Twist1 vector, si-Twist1 (Supplement Fig. 1B,C), miR-22 mimic and inhibitor (Supplement Fig. 1D,E) were assessed by RT-qPCR. Rescue experiment showed that MG63 transfected with miR-22 mimic and Twist1 vector could remedy the reduced proliferation (Fig. 4A–F), migration and invasion (Fig. 5A–E) effective causing by MG63 transfected with miR-22 mimic only. Low expression of Twist1 in MG63 could remedy the raised proliferation (Supplement Fig. 2A–F), migration and invasion (Supplement Fig. 3 A–E) effect causing by MG63 transfected with miR-22 inhibitor only. Twist1 vector could also remedy the effect of EMT signal pathway causing by MG63 transfected with miR-22 mimic only (Supplement Fig. 4). A dual luciferase reporter assay was performed to confirm the binding sites between miR-22 and Twist1. miR-22 overexpression in MG63 cells reduced luciferase activity in cells expressing wild-type Twist1 compared with that in MG63 cells transfected with the mimic control and mutant Twist1 (Supplement Fig. 5). These results showed that miR-22 inhibit MG63 proliferative, migration and invasion through binding Twist1.

### Exosome original MSC could be uptake by MG63

**Figure 6:**
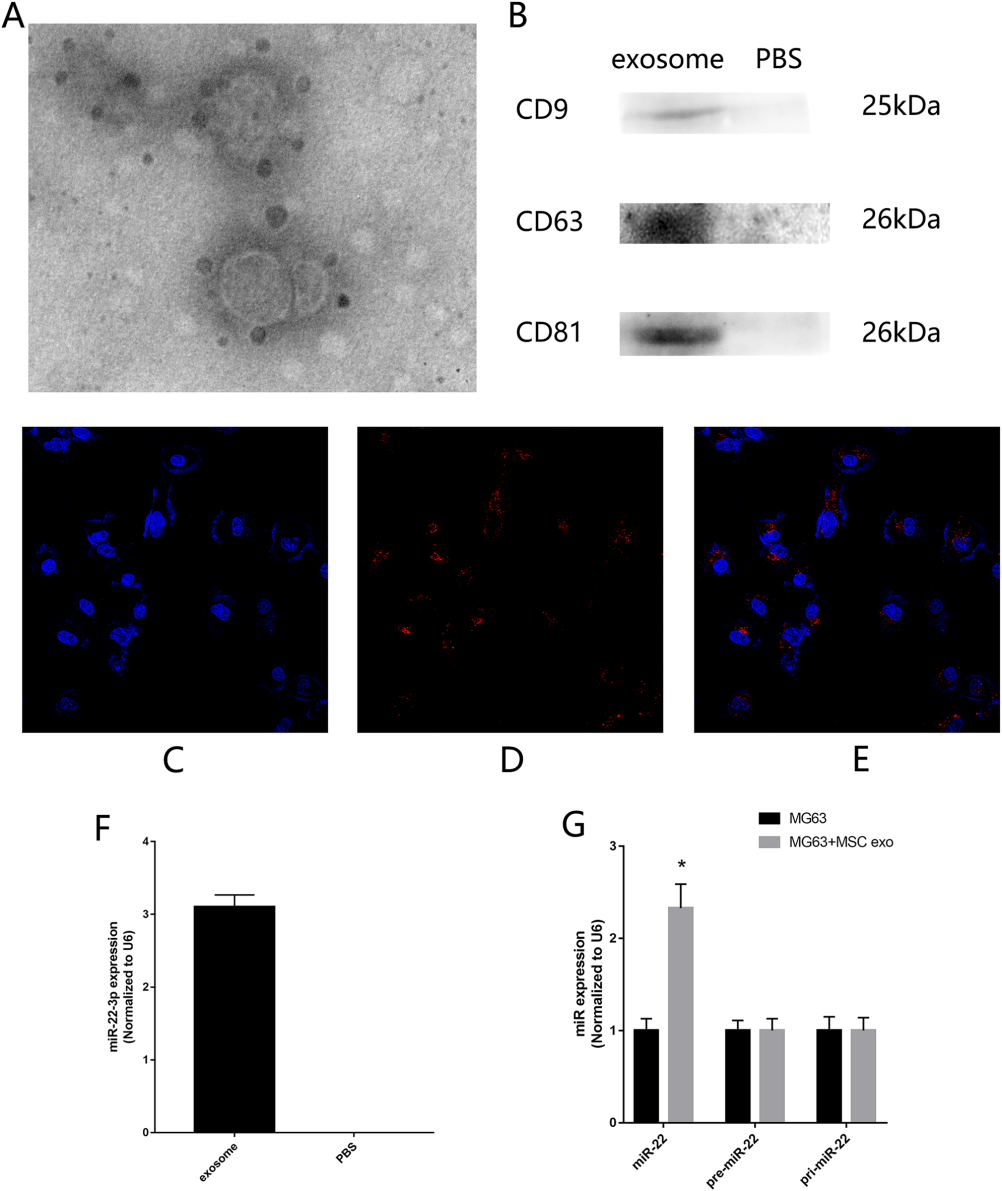
*Exosome original MSC could be uptake by MG63*. (**A**) Electron microscope confirms the size and morphology of exosomes. (**B**) Western blotting confirms the exosomes membrane marker protein such as CD9, CD63, CD81. (**C**–**E**) Confocal microscope. (**F**) RT-qPCR assessed the existence of miR-22 in exo-MSC. (**G**) RT-qPCR assessed the expression of mature miR-22, pre-miR-22, pri-miR-22. (n = 3,*P ≤ 0.05 vs. MG63).

Exosome was acquire from ultrahigh speed centrifugation and verified by transmission electron microscope and western blotting (Fig. 6A,B). After labeling with PKH26, exosomes were cocultured with MG63. Confocal microscope showed that red fluorescence of PKH26-labeled exosomes were observed in MG63 after 2 h coculture (Fig. 6C,E). These results showed that exo-MSC could be uptake by MG63. RT-qPCR showed that miR-22 exist in exo-MSC (Fig. 6F). Then exosomes were added in the MG63 culture medium for 2 h. Mature miR-22, pre-miR-22, pri-miR-22 was detected by RT-qPCR. The results showed that high expression of mature miR-22 was detected in MG63 cultured with exo-MSC. No significant change was found in the expression of pre-miR-22 and pri-miR-22 (Fig. 6G). These results showed that high expression of miR-22 in MG63 originated from exo-MSC.

### Exosome miR-22 original MSC inhibit MG63 proliferative, migration and invasion

Exosomes were added in the MG63 culture medium. MTS assay (Supplement Fig. 6A), CCK-8 assay (Supplement Fig. 6B) and colony formation assay (Supplement Fig. 6C) showed downregulated proliferative of MG63 co-culture with exo-MSC comparing with MG63 co-culture with PBS. RT-qPCR (Supplement Fig. 6D) and western blotting (Supplement Fig. 6E) showed low expression of PCNA in MG63 co-culture with exo-MSC. Exo-MSC reduced cell proliferation, as confirmed by EdU proliferation assays (Supplement Fig. 6F). Transwell assay, wound healing assay showed downregulated migration and invasion of MG63 co-culture with exo-MSC (Supplement Fig. 7). These results showed that exo-MSC inhibit MG63 proliferative, migration and invasion.

**Figure 7:**
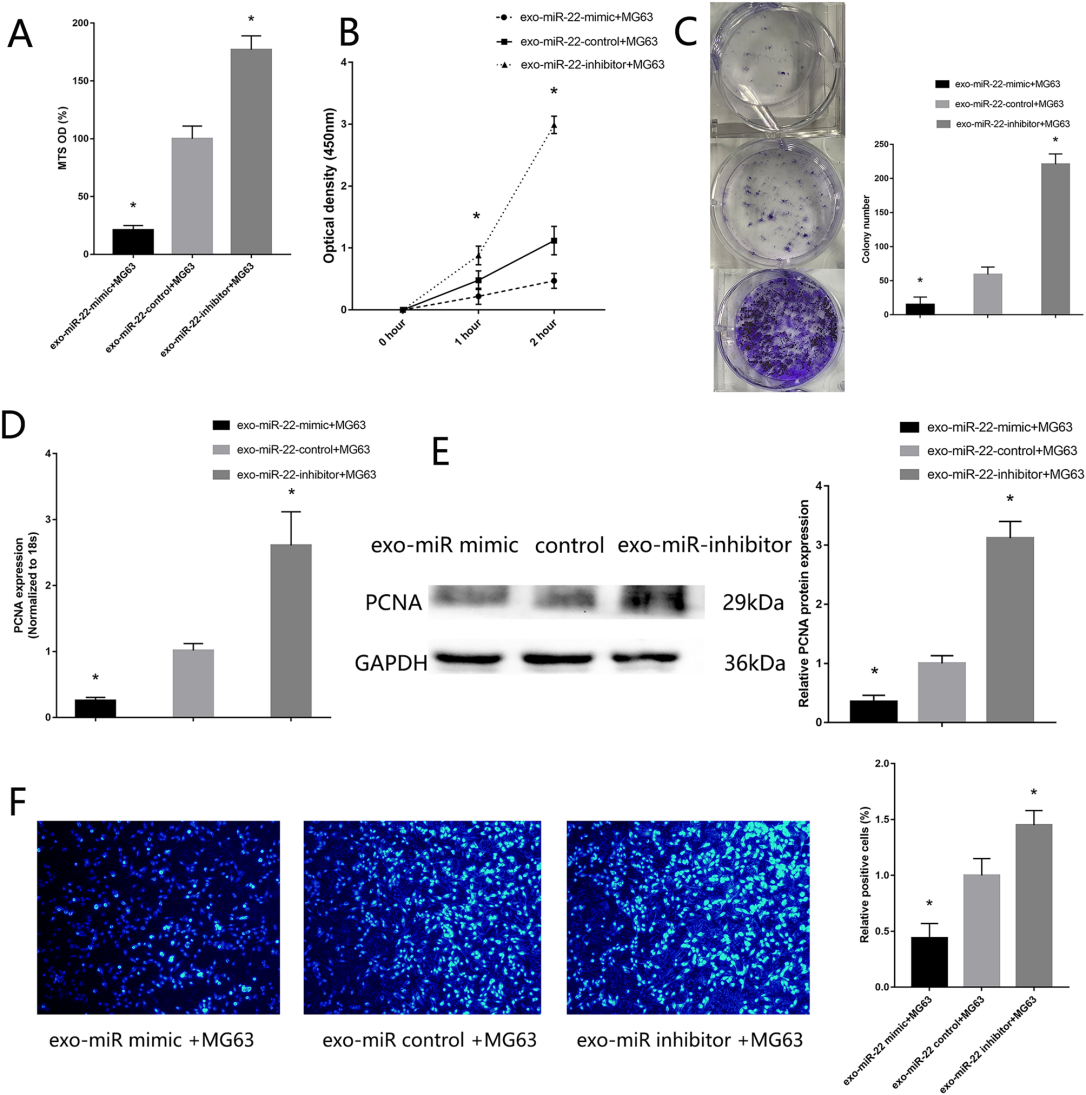
*Exosome miR-22 original MSC inhibit MG63 proliferative*. (**A**) MTS assay detected the proliferation of MG63 co-culture with high-miR22-exo-MSC and low-miR22-exo-MSC. (**B**) CCK-8 assay. (**C**) colony formation assay. (**D**) RT-qPCR detected the expression of PCNA. (**E**) Western blotting detected the expression of PCNA. (F) EdU assays. (n = 3,*P ≤ 0.05 vs. exo-MSC).

**Figure 8:**
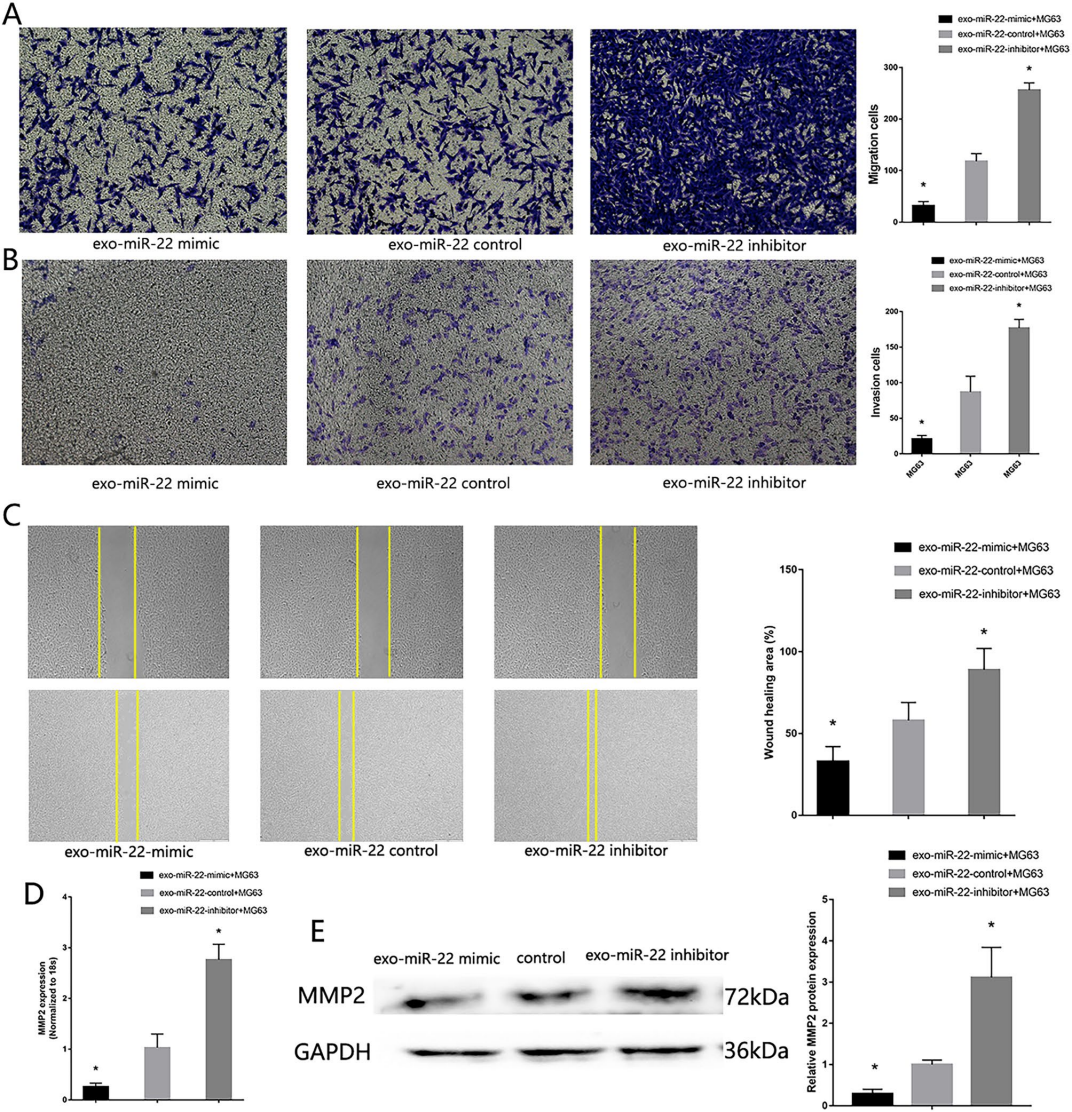
*Exosome miR-22 original MSC inhibit MG63 migration and invasion*. (**A**) Transwell migration assay detected the migration of MG63 co-culture with high-miR22-exo-MSC and low-miR22-exo-MSC. (**B**) Transwell invasion assay detected the invasion of MG63 co-culture with high-miR22-exo-MSC and low expression exosome. (**C**) Wound healing assay. (**D**) RT-qPCR detected the expression of MMP2. (**E**) Western blotting detected the expression of MMP2 (n = 3,*P ≤ 0.05 vs. control).

In order to verify the effect of miR-22 cargo in exo-MSC inhibit MG63 proliferative, migration and invasion, miR-22 mimic and inhibitor were transfected in MSC, which acquire high expression exosomes original MSC (high-miR22-exo-MSC) and low expression exosomes original MSC (low-miR22-exo-MSC). The transfect efficiency was confirmed by RT-qPCR. Then the modified exosomes were added into the MG63. Low proliferative (Fig. 7), migration and invasion (Fig. 8) ability were found in MG63 co-culture with high-miR22-exo-MSC, High proliferative (Fig. 7), migration and invasion (Fig. 8) ability were found in MG63 co-culture with low-miR22-exo-MSC. The functions of miR-22 cargo in exo-MSC in Saos were also verified (Supplement Fig. 8).

## Discussion

In this study, we found that exo-MSC could inhibit MG63 proliferation, migration and invasion. Exosomes original from MSC contain proteins, lipids, RNA, metabolites, growth factors and cytokines, which had a function on promoting healing and regeneration, inhibiting inflammation^19^. Exosomes have become as necessary regulators during intercellular communication, which control many physiological and pathological processes in different disease. MSC could inhibit tumor progression through the paracrine exosomes. Therefore, exo-MSC may be a cell-free cancer treatment alternative for further gene target therapy.

MiRNA is one of the main cargo in exo-MSC. It is an evolutionary conservative gene and exists in tissues, cells and body fluid. MiR-22 is reported to regulate various physiological and pathological processes. It could target and inhibit downstream transcription factors and other target genes, which largely determining the fate of cancer. MiR-22 could act as a tumor suppressor miRNA, which is abnormally down-regulated in prostate cancer^20^, esophageal squamous cell carcinoma, cervical cancer^20^, breast cancer^21^ and gastric cancer^16^. It can also act as a tumor a carcinogenic miRNA that exacerbates cancer formation and malignant transformation such as cutaneous squamous cell carcinoma^22^, hepatocellular carcinoma^23^. In addition, the report also indicates that miR-22 may significantly affect the biological behavior of cancer, such as proliferation, invasion and metastasis, and it genetically alters the expression of many related genes. For example, miR-22 suppresses cell proliferation, migration, and invasion in colorectal cancer by targeting NLRP3^24^. MiR-22 downregulated the expression of NK1R-Tr and ERα to delay and weaken phosphorylation of ERK1/2 to inhibit proliferation and metastasis of breast cancer cells^25^.

Recently, the roles of miR-22 on proliferation, invasion and metastasis are also found in the OS. Due to the complexity of the regulatory mechanisms of miR-22, the potential clinical applications of miR-22 in regulating OS progression have not been elucidated. In this study, bio-informatic analysis showed that miR-22/Twist1/CADM1 might regulate the OS proliferation, invasion, and metastasis. RT-qPCR showed that low expression of miR-22 in OS tissues and MG63 and Saos. Because of the expression of miR-22 reduced larger in MG63 comparing with that reduced in Saos, we choose MG63 for the following experiments. Then we test if exo-MSC could carry large of miR-22 and transmit miR-22 to MG63. We label exo-MSC with PKH-26 (red) and added them into the MG63 culture medium. The red label exo-MSC were observed in the cytoplasm of MG63, which proved that MG63 could uptake exo-MSC.

Actually many growth factors, cytokines, protein and genes cargo in exo-MSC, which could regulate the fate of MG63. In order to verify the function of miR-22 cargo in exo-MSC on MG63 proliferation, invasion and metastasis, we modified exo-MSC with miR-22 mimic and inhibitor. Low proliferative, migration and invasion ability were found in MG63 co-culture with high-miR22-exo-MSC, comparing with MG63 co-culture with exo-MSC. Therefore, the miR-22 in exo-MSC could be uptake by MG63 and inhibit MG63 proliferation, migration and invasion.

Then we explore the molecular mechanism of miR22 after uptake by MG63. MiRNA typically exerts its biological function by binding the 3′ UTR segment of downstream target genes. Twist1 is one of the target genes of miR22^17^. The Twist1 gene is located on chromosome 7p21 which contains two exons and one intron. Twist1 is an important transcription factor belonging to the evolutionary conserved basic helix family. The post translational modifications of Twist family proteins, especially the phosphorylation of PKA, PKB, CK2, MAPK, regulate the function of Twist proteins. At the same time, Twist1 is also regulated by miRNA, including transcriptional regulation of chromatin conformation and methylation of Twist1 promoter region, regulating the role of Twist1 in physiological or pathological processes. At present, the expression of Twist1 is abnormally high in many tumors, such as breast cancer, prostate cancer and hepatocellular carcinoma. Twist1 plays multiple roles in cancer, affecting the occurrence, progression, and metastasis of tumors. Targeted Twist1 or Twist1 related molecules has been shown to be a promising cancer treatment method. In this study, combine the bio-informatics analysis and verification experiments, we found a consistent trend of the changing between CADM1 and miR-22. Therefore, we infer that some medium may exist between CADM1 and miR-22. Twist1 was a transcription factor, which had potential binding sites with miR-22. we used a dual luciferase reporter assay to confirm the effect of miR-22 on Twist1, which prove the binding sites between miR-22 and Twist1. Then we used Twist1 plasmid and si-Twist1 for rescue experiments, which showed high expression of Twist1 in MG63 transfected with miR-22 mimic and Twist1 vector could remedy the reduced proliferation and migration and invasion effect causing by MG63 transfected with miR-22 mimic only. Therefore, after uptake by MG63, miR-22 cargo in exo-MSC could enter MG63 cytoplasm, and inhibit MG63 proliferation, migration and invasion through targeting Twist1.

In conclusion, miR-22 was downregulated in OS patients and MG63 cell line, which promoted MG63 proliferation, migration and invasion through targeting Twist1. miR-22 cargo in exo-MSC could uptake by MG63 and supply MG63 with miR-22, which inhibit MG63 proliferation, migration and invasion through targeting Twist1. These results might provide novel mechanistic insight into the pathogenesis of OS and serve as a promising diagnostic biomarker and cell-free therapeutic method for the treatment of OS.

### Supplementary Information

Supplementary Figures.

## Data Availability

The data used to support the findings of this study are available from the corresponding author upon request.

## Abbreviations

miRNAs: MicroRNAs
RT-qPCR: Reverse transcription-quantitative PCR
CCK: Cell counting kit
MSC: Mesenchymal stem cells
OS: Osteosarcoma
Exo-MSC: Mesenchymal stem cell exosomes
EMT: Epithelial mesenchymal transition
