# Supplementary material for: Exosome microRNA-22 inhibiting proliferation, migration and invasion through regulating Twist1/CADM1 axis in osteosarcoma
Source: Sci Rep. 2024 Jan 8;14:761. doi: 10.1038/s41598-023-50612-4 (PMC10774347; doi:10.1038/s41598-023-50612-4)

Supplementary Figure 1

(A) The transfection efficiency of miR-22 assessed by RT-qPCR. (B, C) The transfection efficiency of Twist1 assessed by RT-qPCR and western blotting. (D, E) The transfection efficiency of Twist1 and miR-22 assessed by RT-qPCR and western blotting. n=3,* P≤0.05 vs. control.


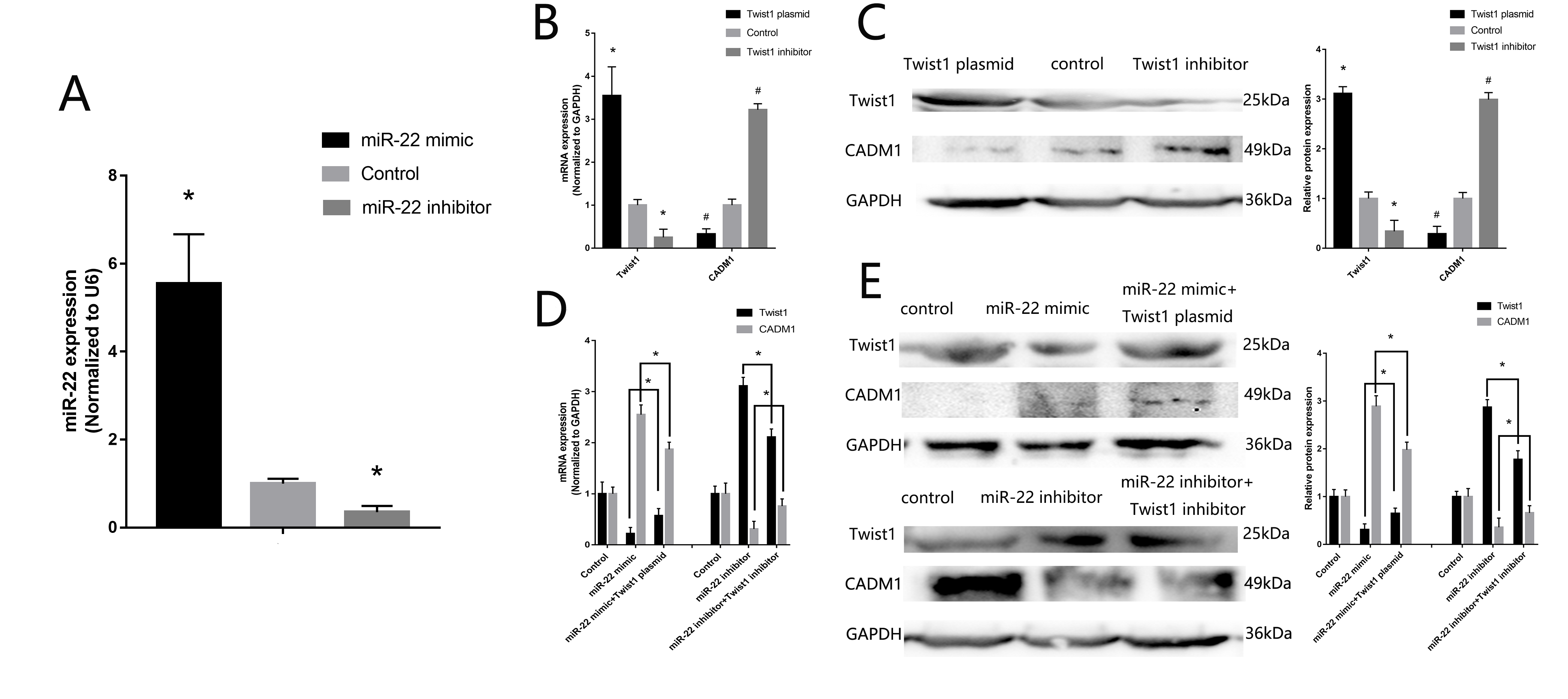


Supplementary Figure 2

***miR-22/Twist1 regulates MG63 proliferative*.** (A) MTS assay detected the proliferation of MG63 transfected with both miR-22 inhibitor and si-Twist1. (B) CCK-8 assay. (C) colony formation assay. (D) RT-qPCR detected the expression of PCNA. (E) Western blotting detected the expression of PCNA. (F) EdU assays. (n=3,* P≤0.05 vs. control).


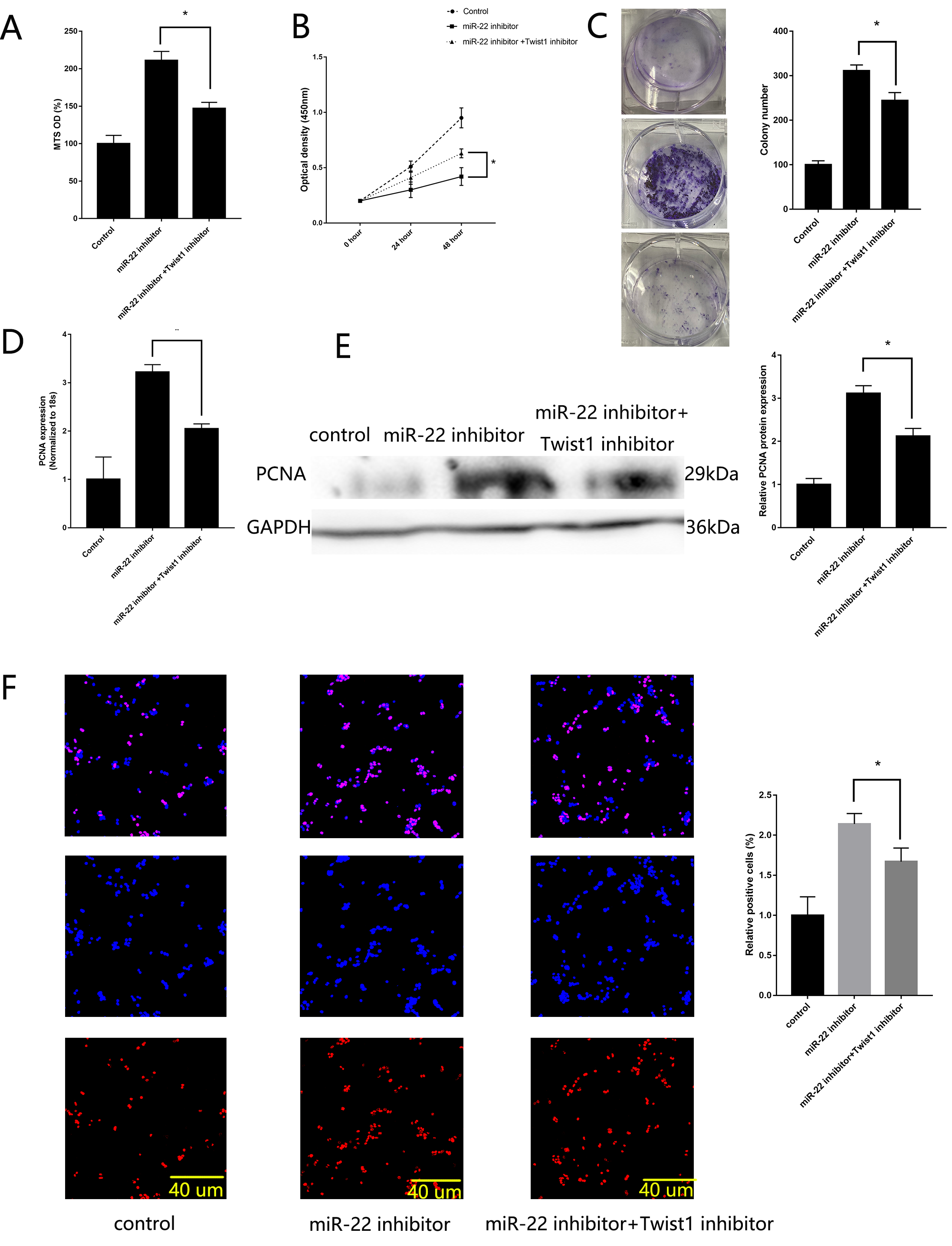


Supplementary Figure 3

***miR-22/Twist1 regulates MG63* *migration and invasion*.** (A) Transwell migration assay detected the migration of MG63 transfected with both miR-22 inhibitor and si-Twist1. (B) Transwell invasion assay detected the invasion of MG63 transfected with both miR-22 inhibitor and si-Twist1. (C) Wound healing assay. (D) RT-qPCR detected the expression of MMP2. (E) Western blotting detected the expression of MMP2 (n=3,* P≤0.05 vs. control).


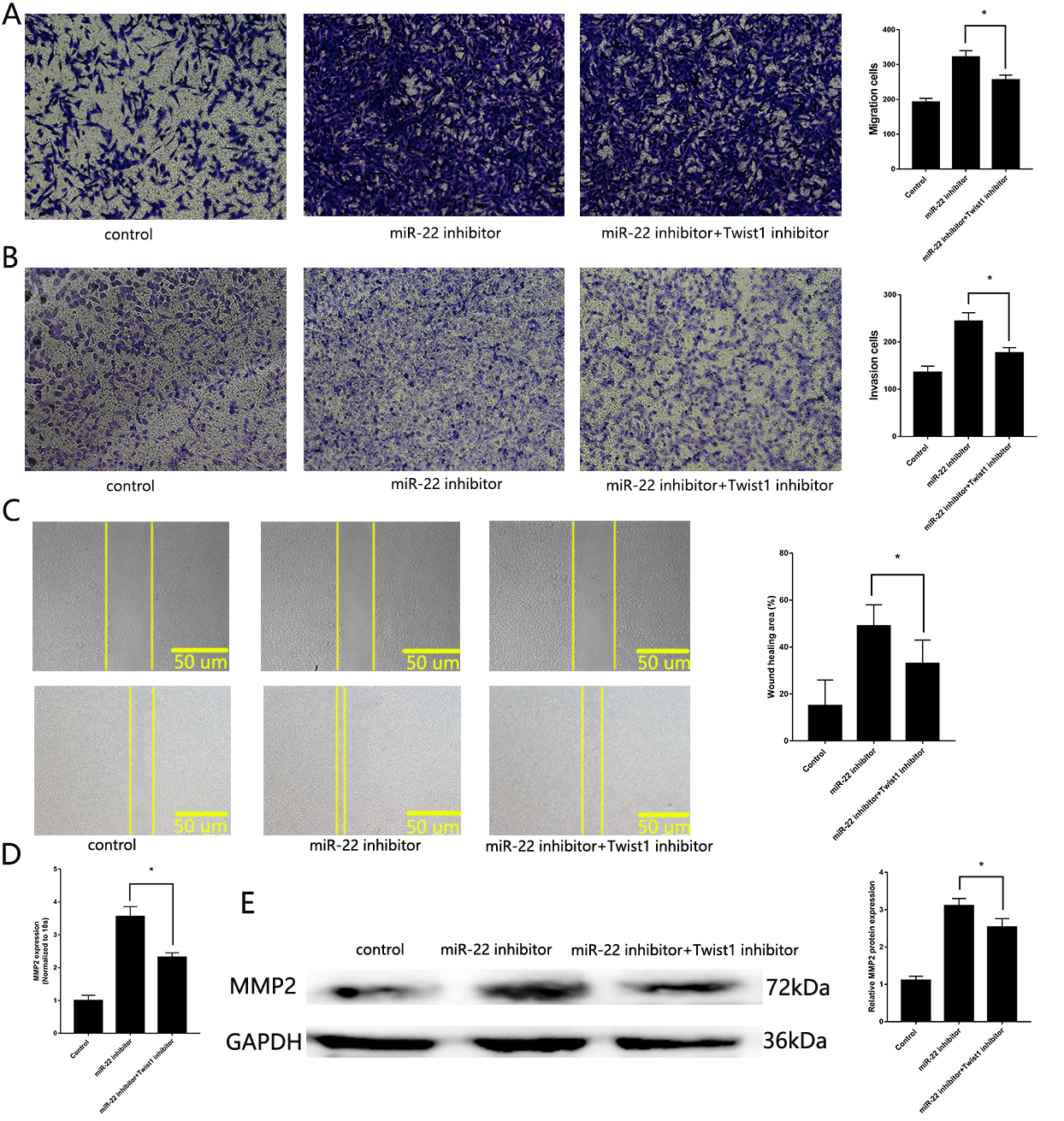


Supplementary Figure 4

The rescue experiment of Twist1 assessed by western blotting. n=3,* P≤0.05 vs. miR-22 mimic or miR-22inhibitor.


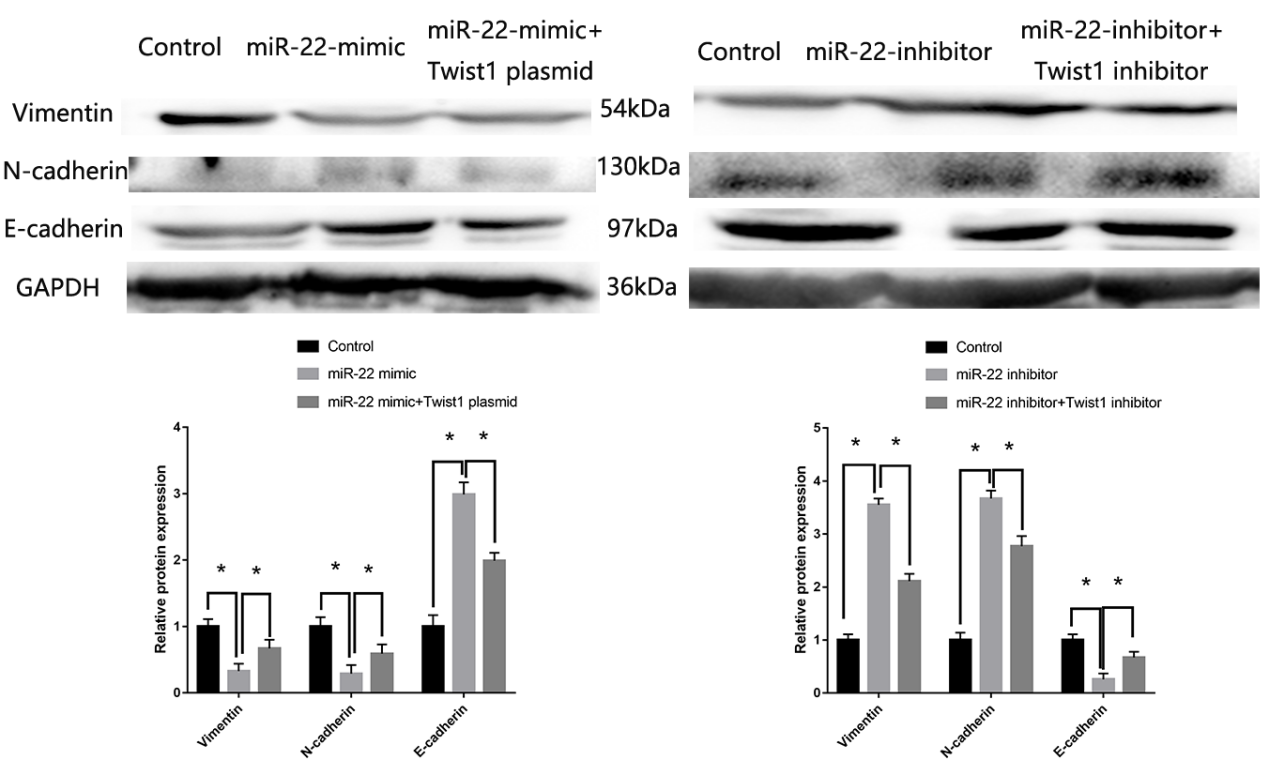


Supplementary Figure 5

Dual luciferase reporter assay assessed the binding sites between miR-22 and Twist1.


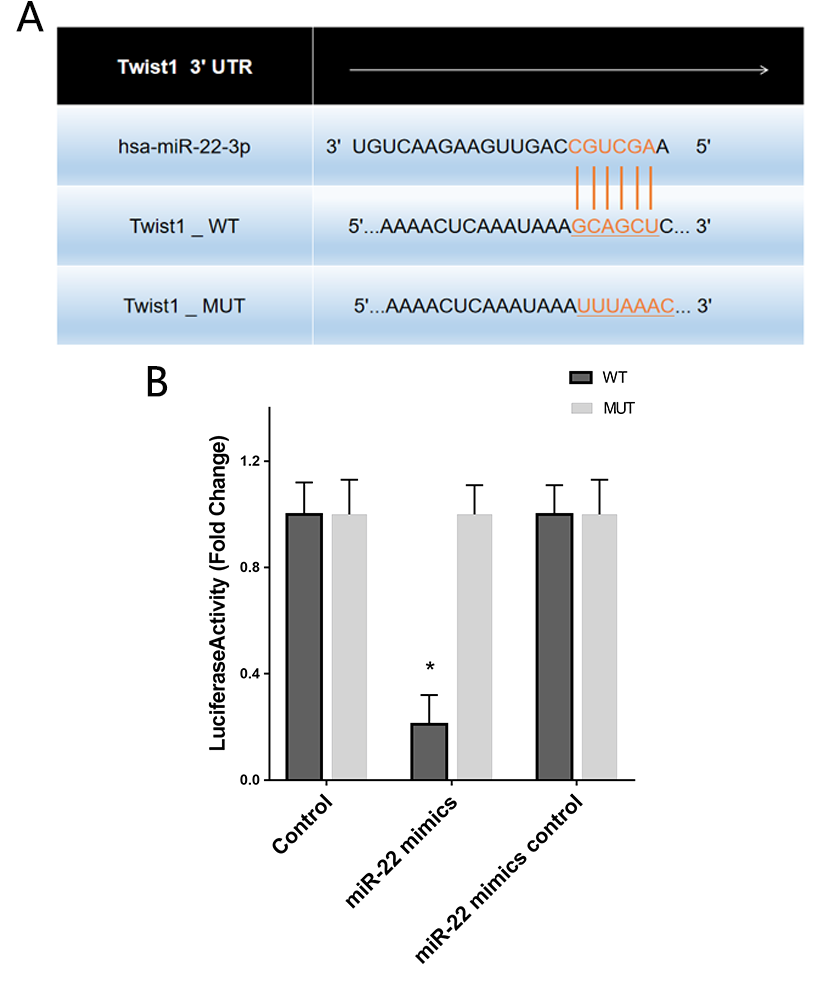


Supplementary Figure 6

***Exo-MSC regulates MG63 proliferative.*** (A) MTS assay detected the proliferation of MG63 co-culture with exo-MSC. (B) CCK-8 assay. (C) colony formation assay. (D) RT-qPCR detected the expression of PCNA. (E) Western blotting detected the expression of PCNA. (F) EdU assays. (n=3,* P≤0.05 vs. control).


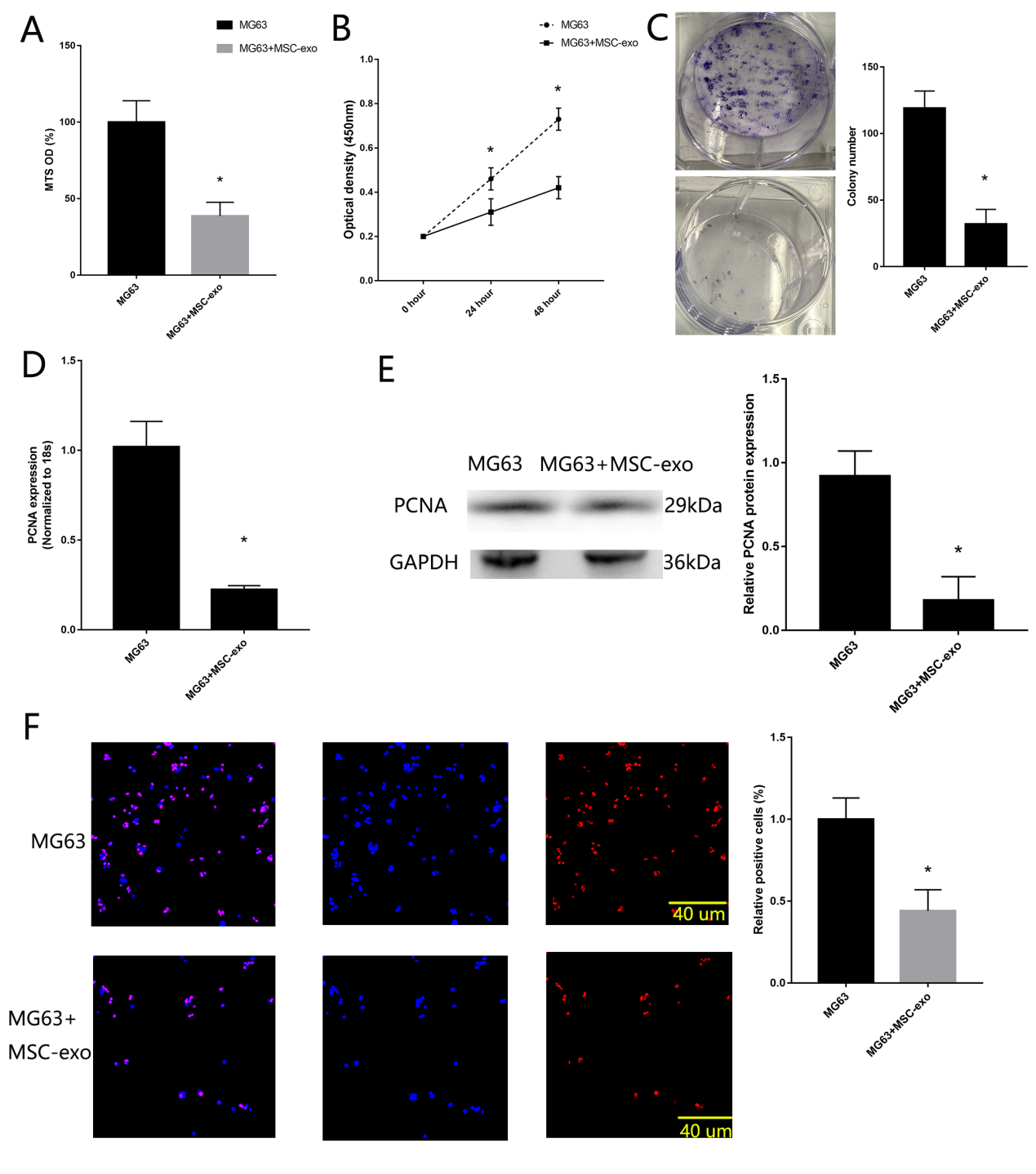


Supplementary Figure 7

***Exo-MSC regulates MG63 migration and invasion.*** (A) Transwell migration assay detected the migration of MG63 co-culture with exo-MSC. (B) Transwell invasion assay detected the invasion of MG63 co-culture with exo-MSC. (C) Wound healing assay. (D) RT-qPCR detected the expression of MMP2. (E) Western blotting detected the expression of MMP2 (n=3,* P≤0.05 vs. control).


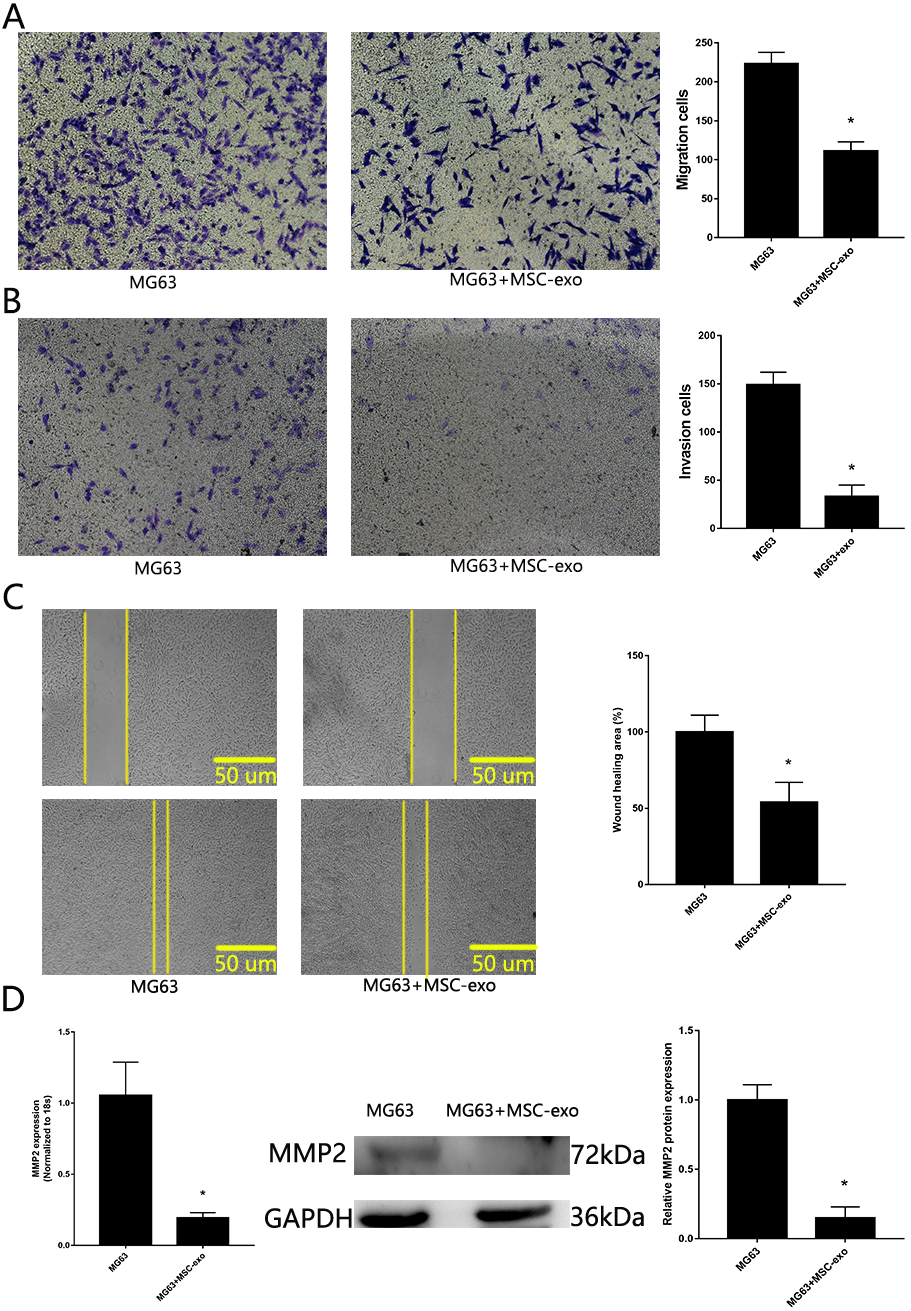


Supplementary Figure 8

***Exo-MSC miR-22 regulates Saos migration and invasion.*** (A) MTS assay detected the proliferation of Saos. (B) colony formation assay. (C) CCK-8 assay. (D) EdU assays. (E) RT-qPCR detected the expression of PCNA. (F) Western blotting detected the expression of PCNA. (G) Wound healing assay. (H) Transwell migration assay. (I) Transwell invasion assay. (J) RT-qPCR detected the expression of MMP2. (K) Western blotting detected the expression of MMP2 (n=3,* P≤0.05 vs. control).


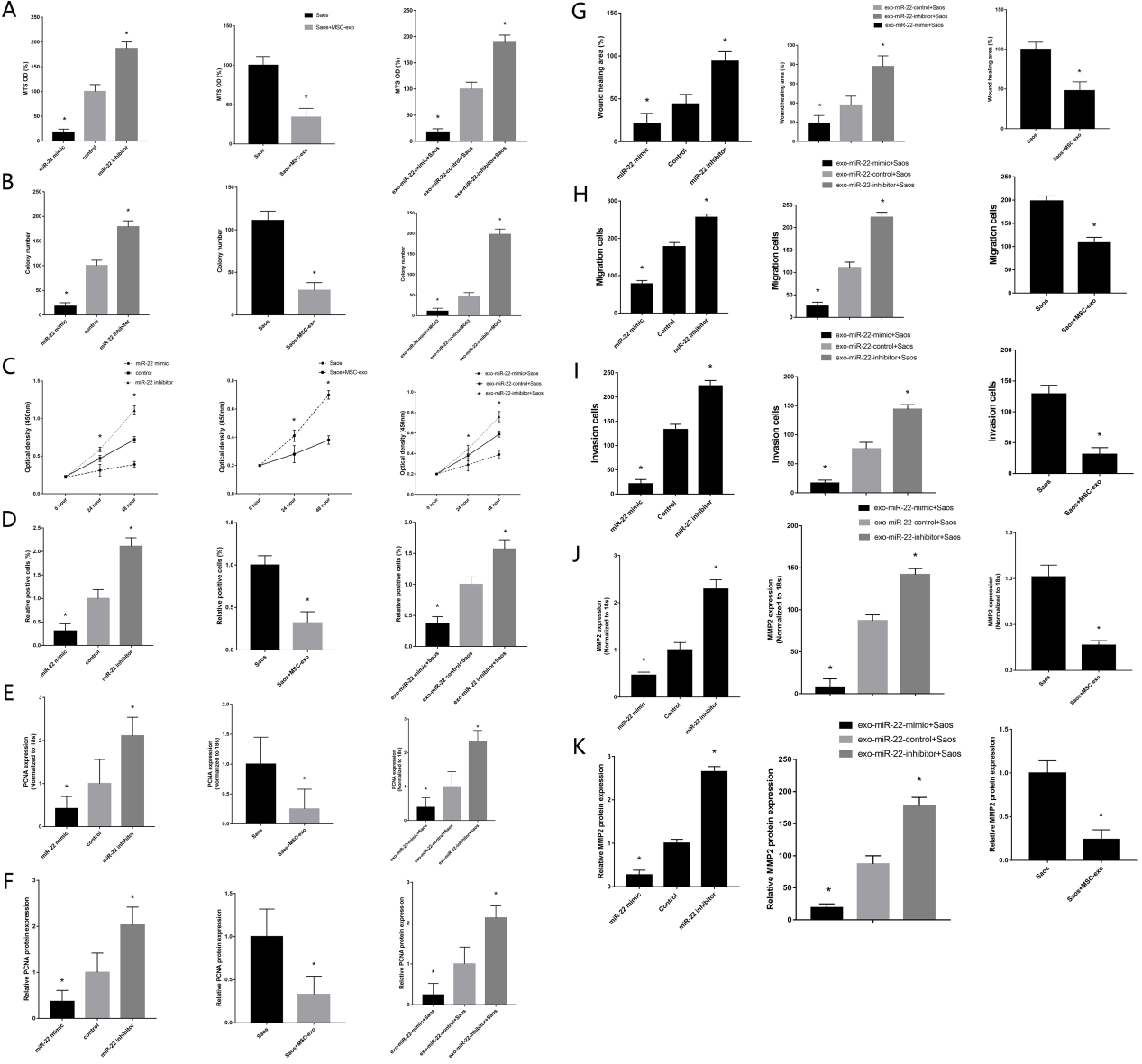


Supplementary raw data of western blotting

Fig.2

GAPDH:
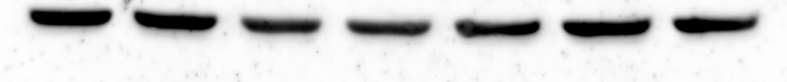


PCNA:
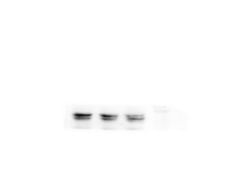


Fig.3

MMP:
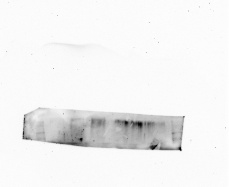


GAPDH:
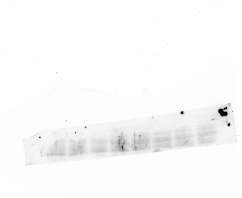


Fig.4

PCNA:
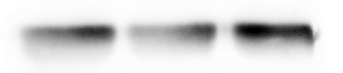


GAPDH:
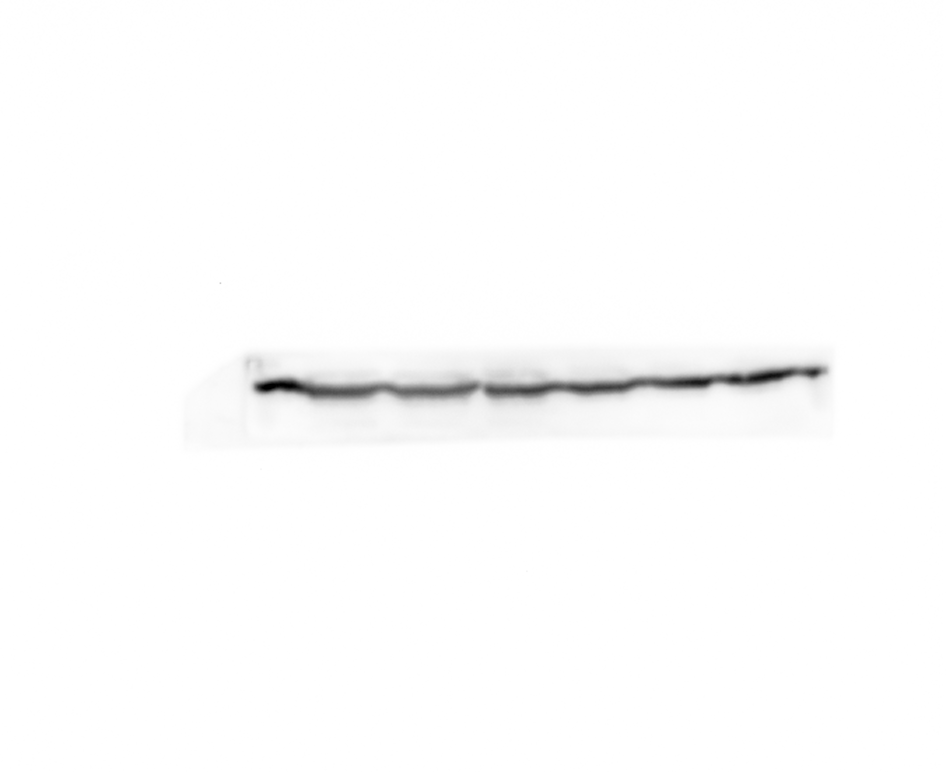


Fig.5

MMP:
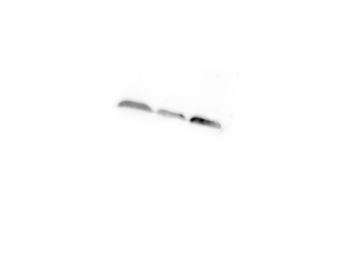


GAPDH:
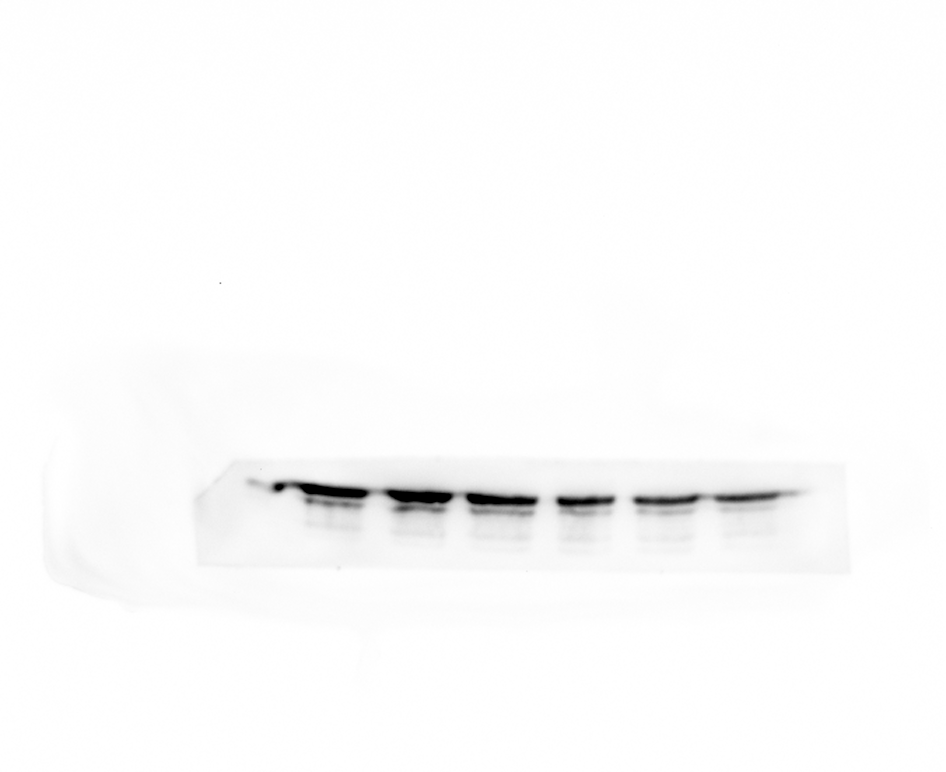


Fig.6

CD9:
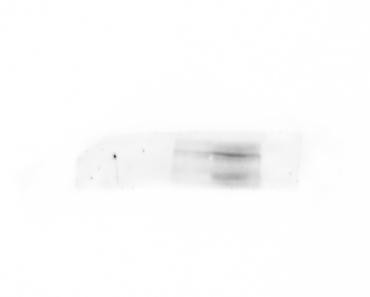


CD63:
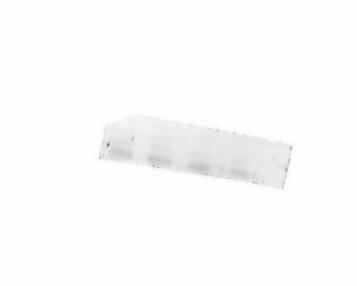


CD81:
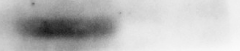


Fig.7:

PCNA:
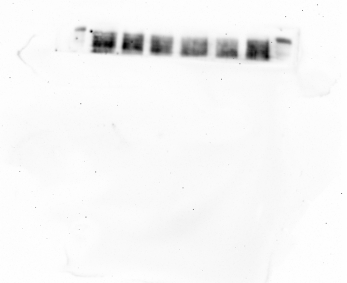


GAPDH:
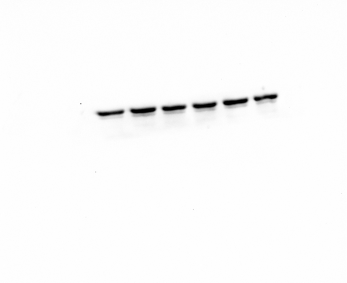


Fig.8

MMP:
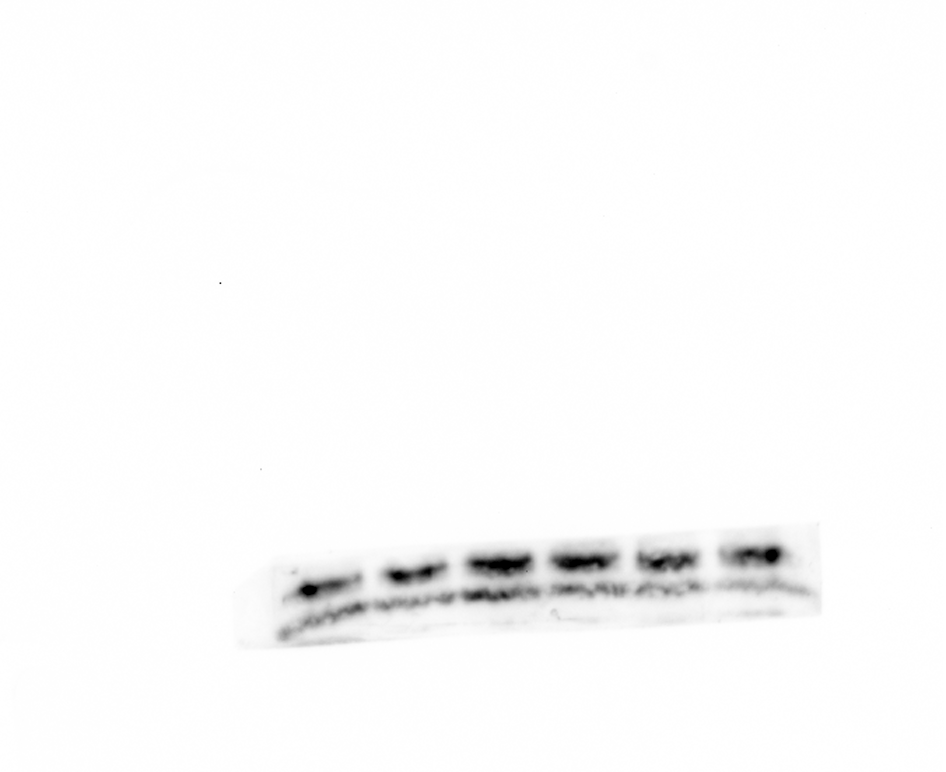


GAPDH:
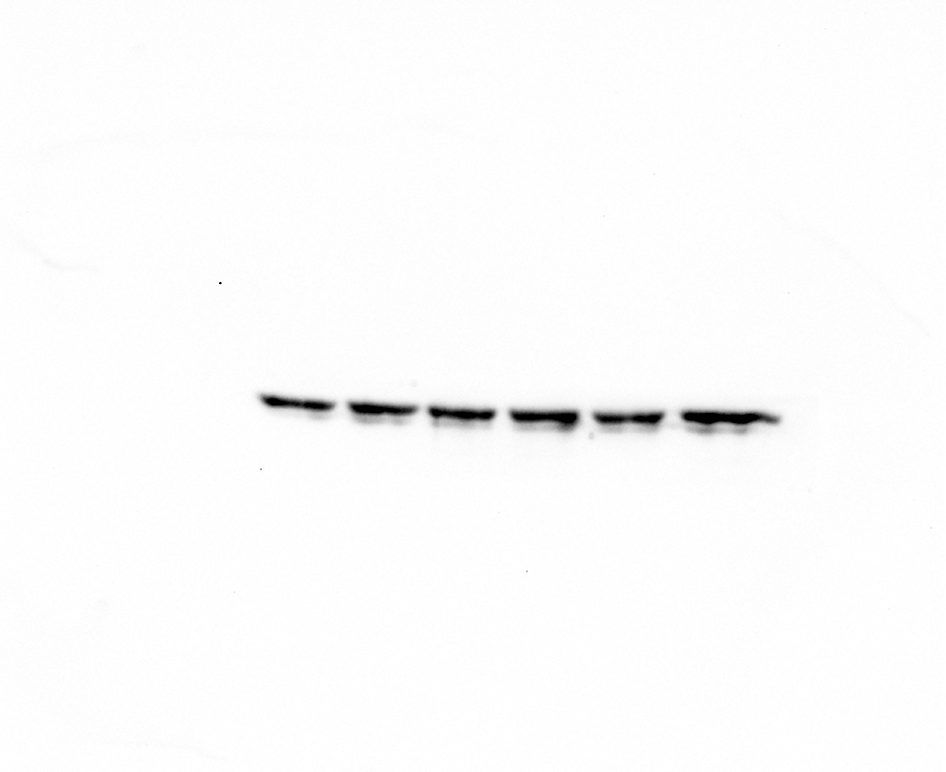


Supplement figure 1C

CADM1:
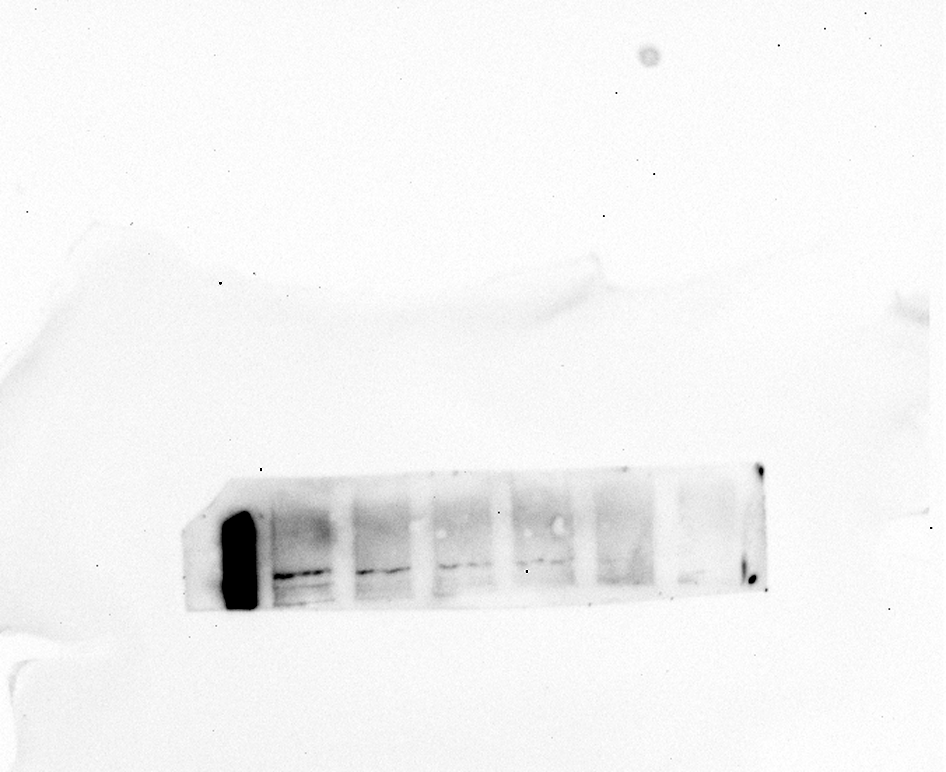


TWIST:
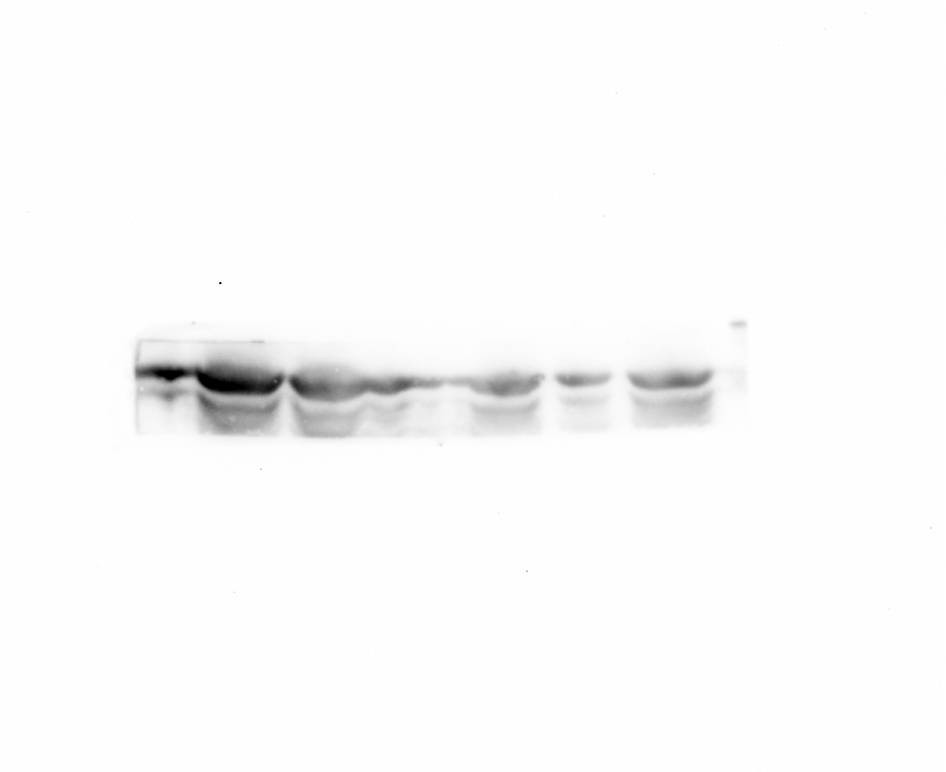


GAPDH:
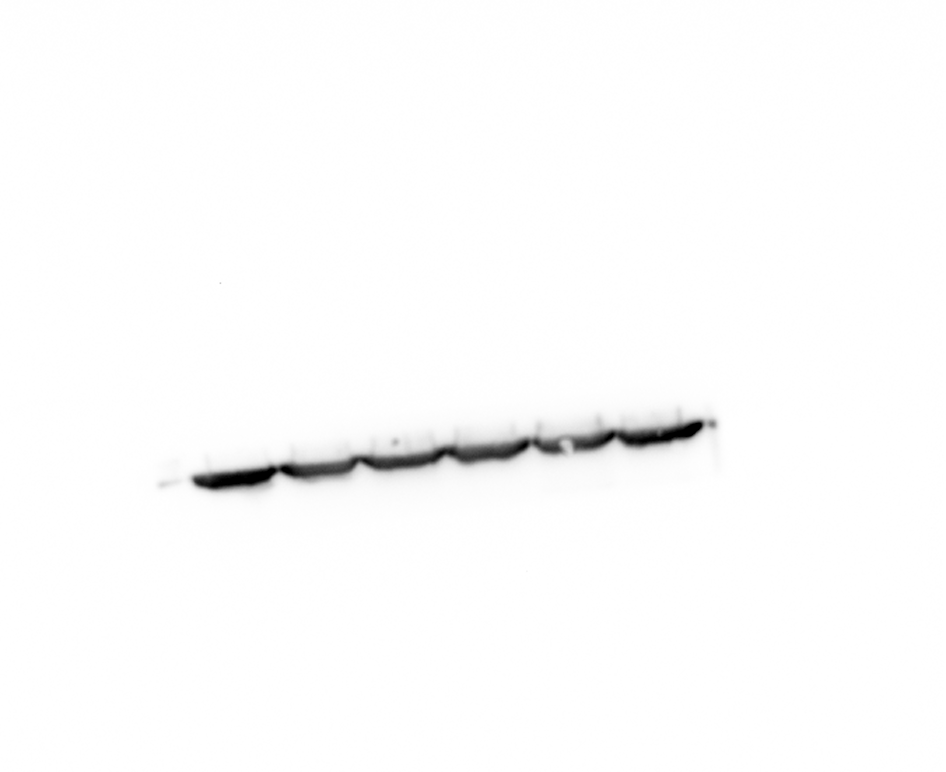


Supplement figure 1E(MG63)

CADM1:
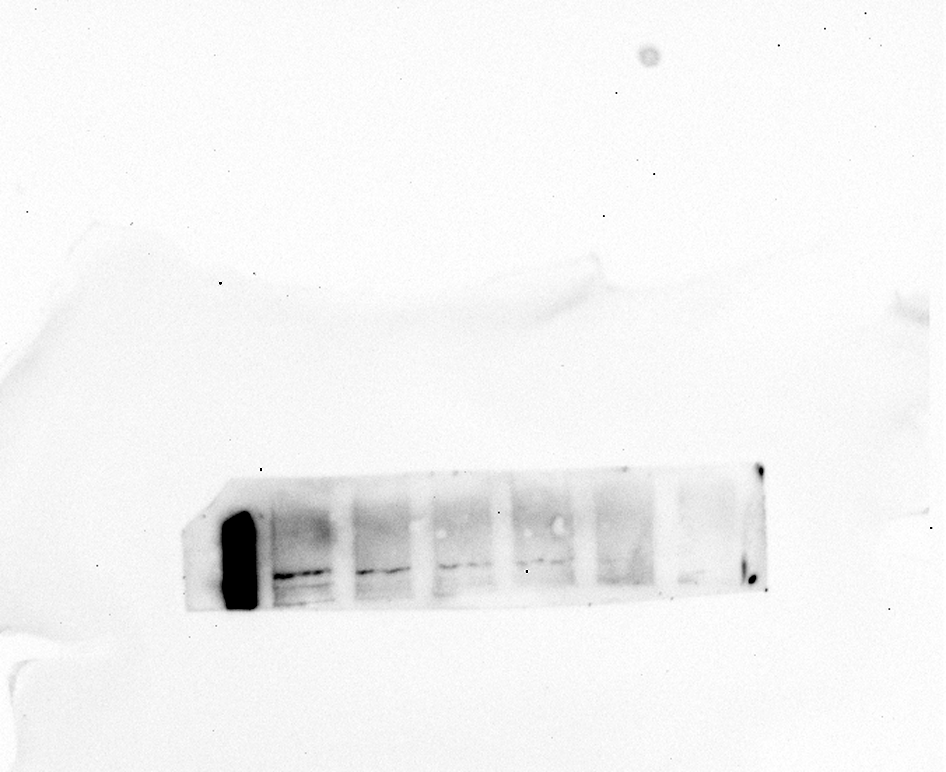


TWIST:
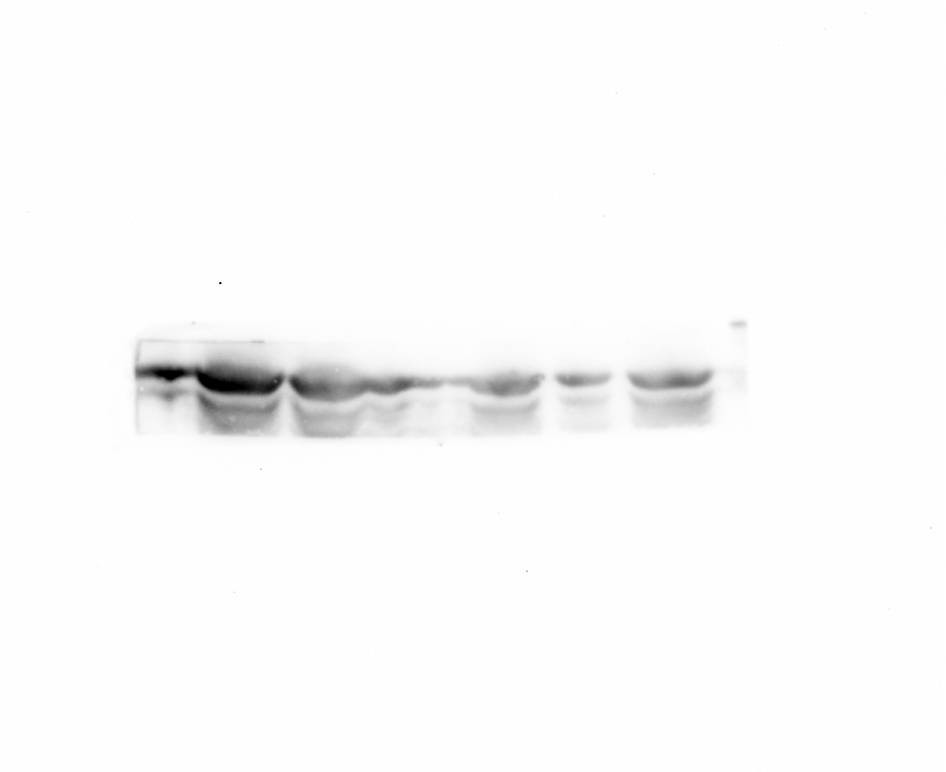


GAPDH:
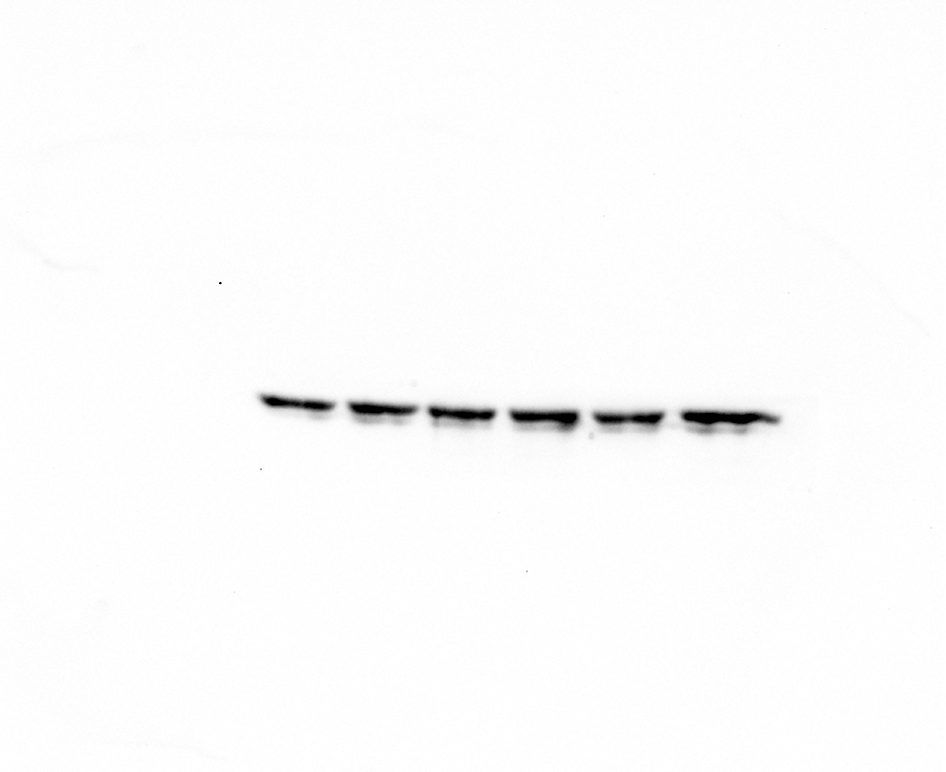


Supplement figure 1E(Saos)

CADM1:
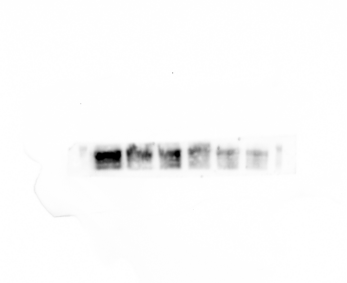


TWIST:
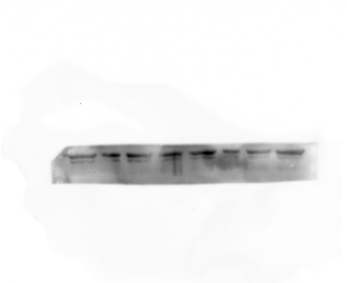


GAPDH:
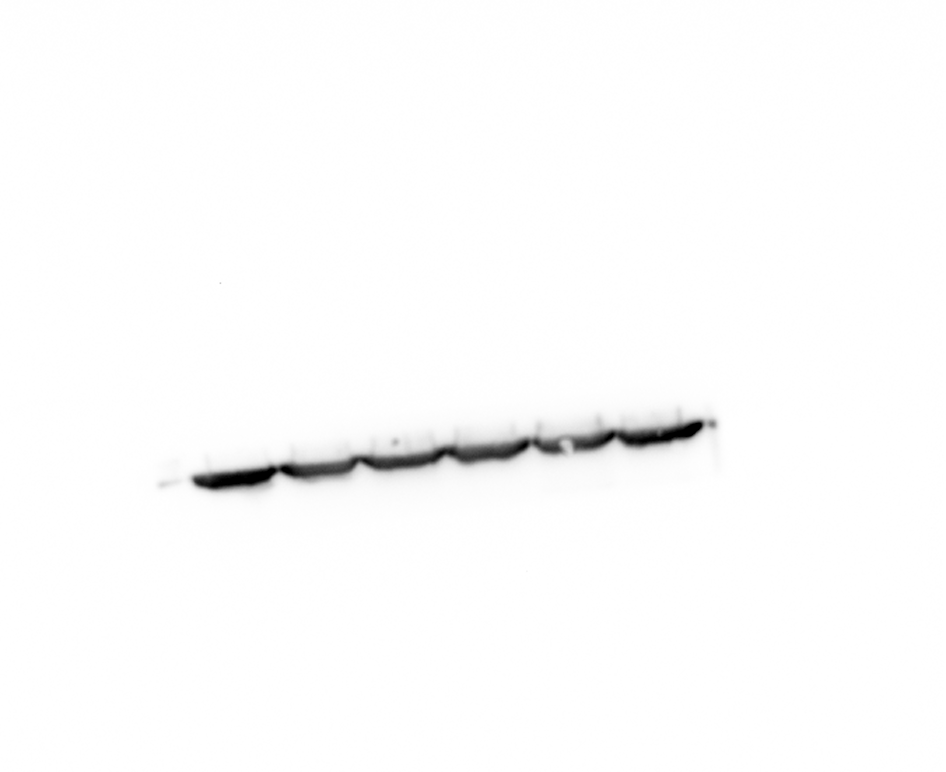


Supplement figure 2

PCNA:
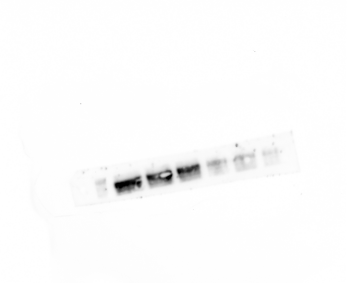


GAPDH:
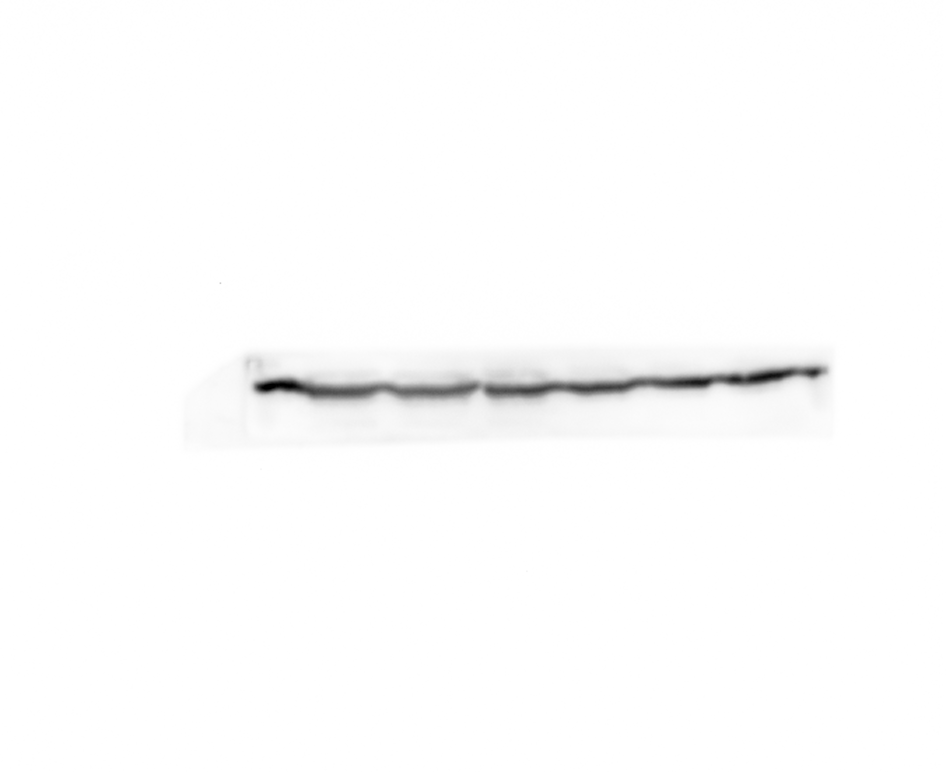


Supplement figure 3

MMP:
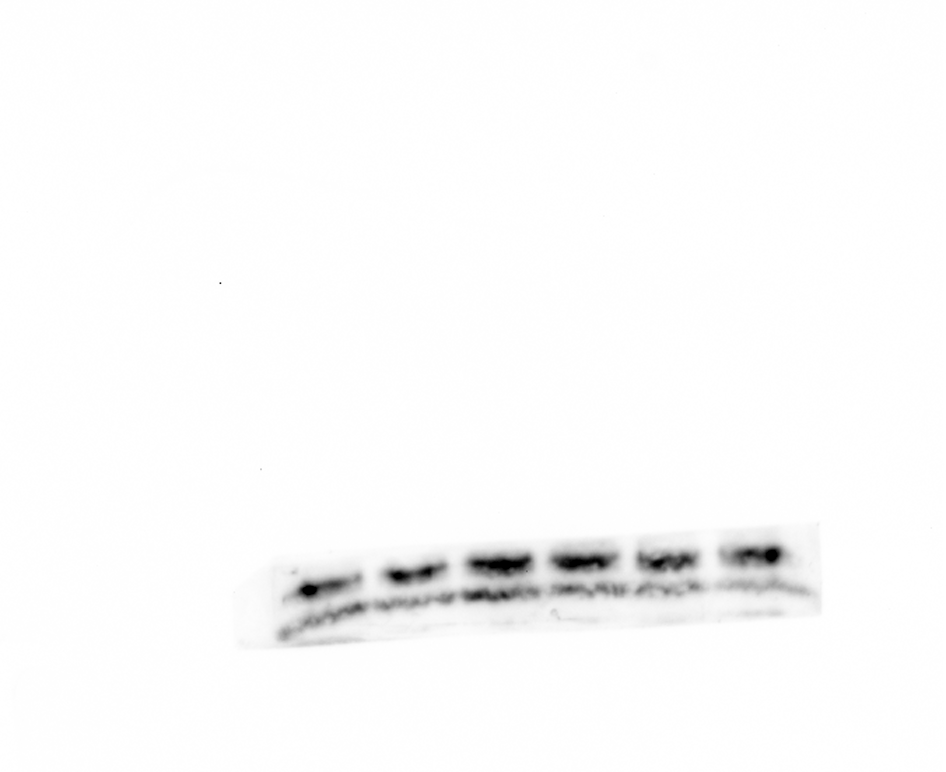


GAPDH:
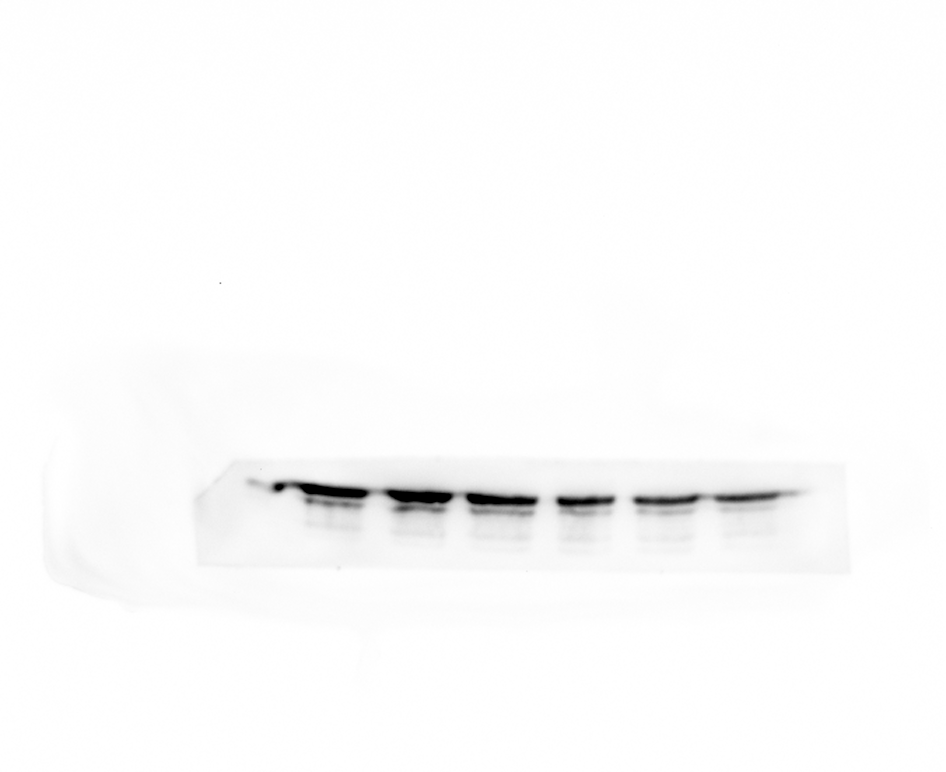


Supplement figure 4

E-CAD:
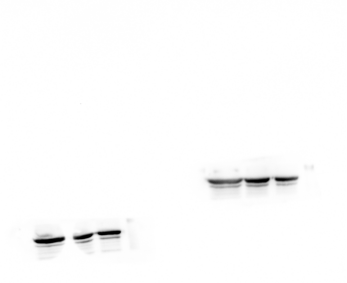


N-CAD:
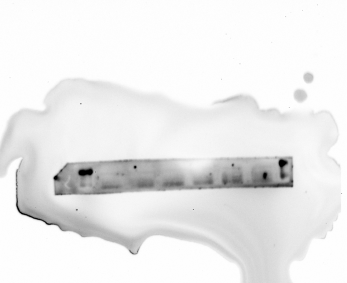


VIMENTIN:
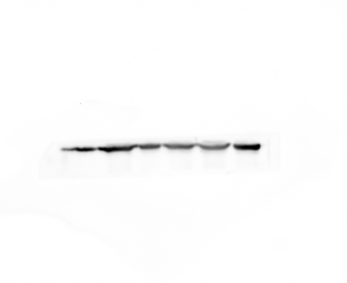


GAPDH:
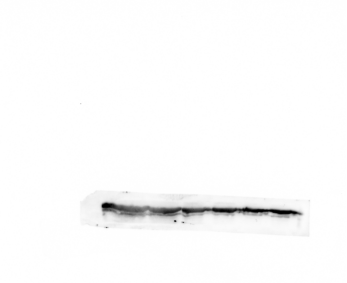


Supplement figure 6

PCNA:
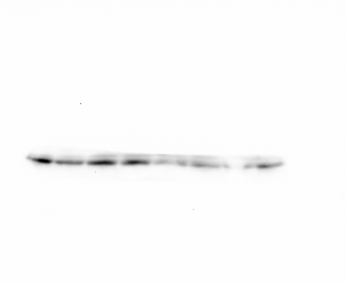


GAPDH:
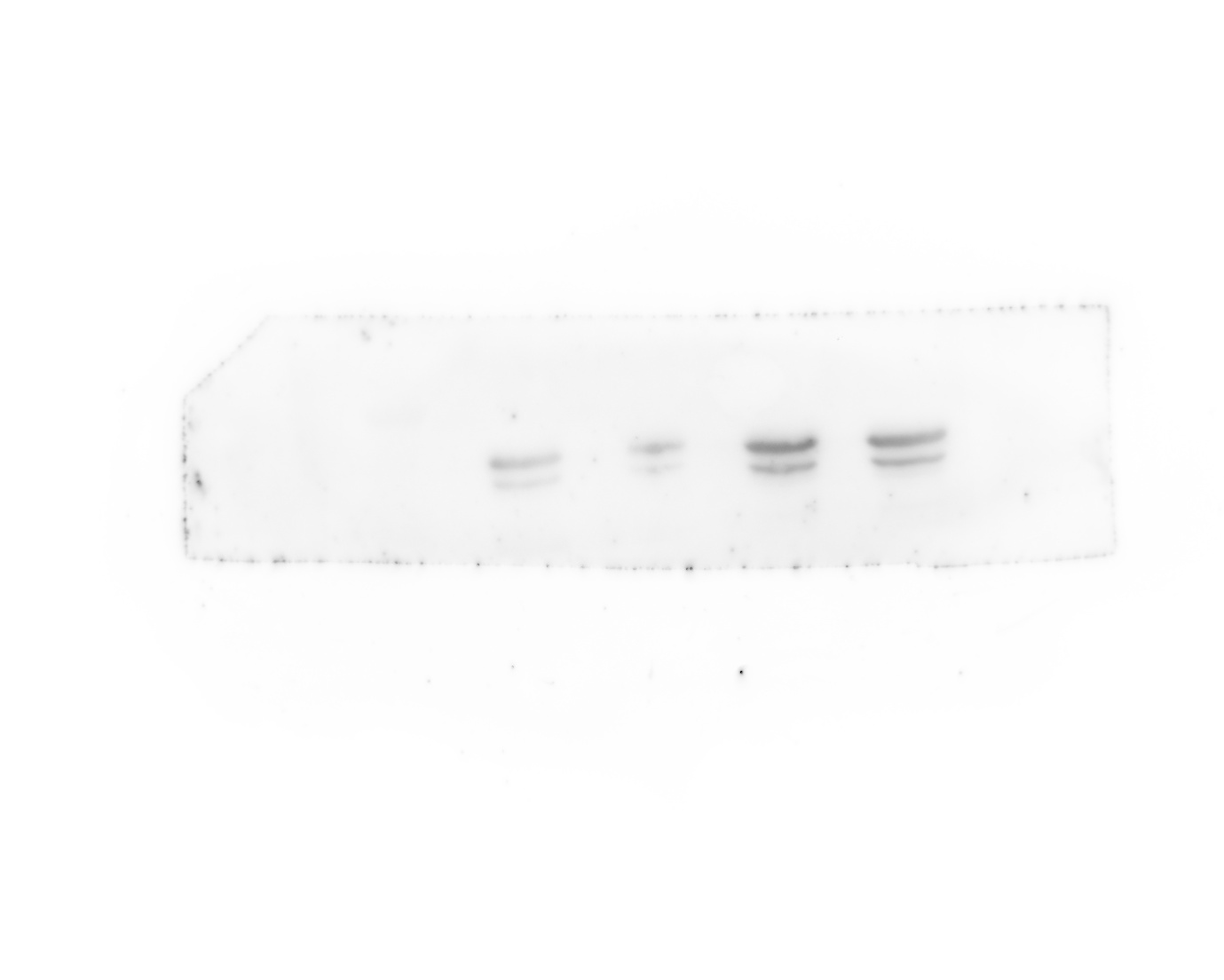


Supplement figure 7

MMP:
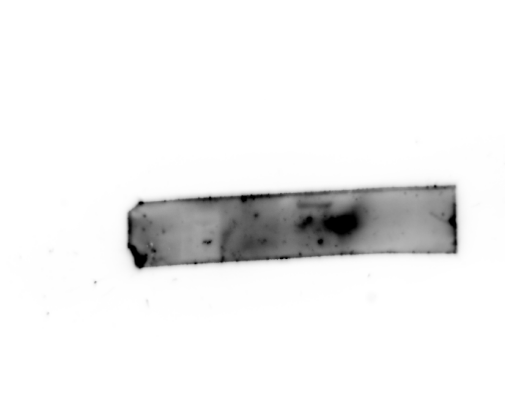


GAPDH:
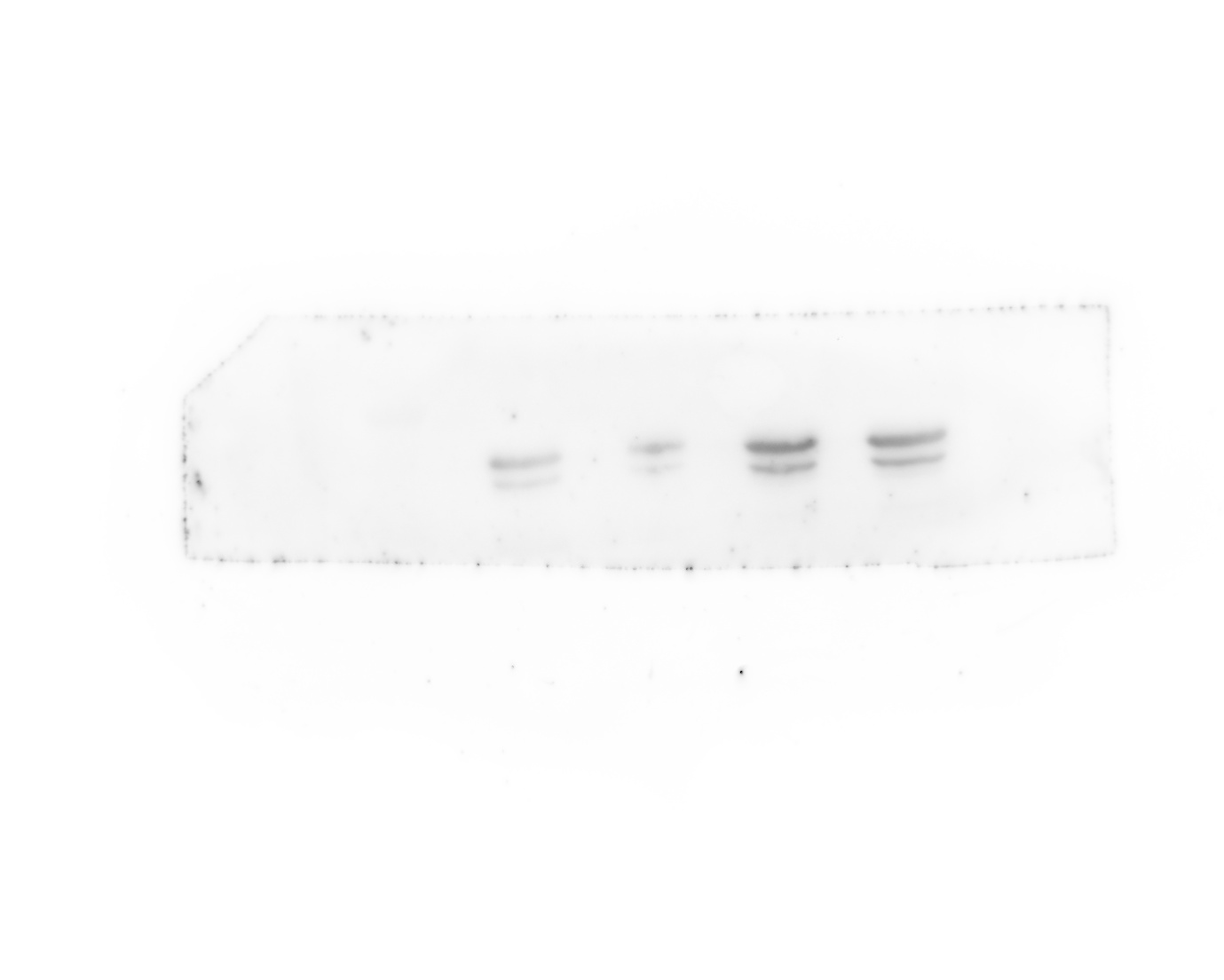

Supplement: Supplementary file 1 — Supplementary Figures. [file 41598_2023_50612_MOESM1_ESM.docx]
